# IGF1R signaling induces epithelial-mesenchymal plasticity via ITGAV in cutaneous carcinoma

**DOI:** 10.1186/s13046-024-03119-3

**Published:** 2024-07-29

**Authors:** Marta Lopez-Cerda, Laura Lorenzo-Sanz, Victoria da Silva-Diz, Sandra Llop, Rosa M. Penin, Josep Oriol Bermejo, Richard de Goeij-de Haas, Sander R. Piersma, Thang V. Pham, Connie R. Jimenez, Juan Martin-Liberal, Purificación Muñoz

**Affiliations:** 1 Oncobell Program, Bellvitge Biomedical Research Institute (IDIBELL), 08908 L’Hospitalet de Llobregat, Barcelona Spain; 2 Medical Oncology Department, Catalan Institute of Oncology (ICO) L’Hospitalet, 08908 L’Hospitalet de Llobregat, Barcelona Spain; 3 Pathology Service, Bellvitge University Hospital/IDIBELL, 08908 L’Hospitalet de Llobregat, Barcelona Spain; 4 Plastic Surgery Unit, Bellvitge University Hospital/IDIBELL, 08908 L’Hospitalet de Llobregat, Barcelona Spain; 5 OncoProteomics Laboratory, Department of Medical Oncology, Amsterdam UMC, 1081HV Amsterdam, the Netherlands; 6 Present Address: Rutgers Cancer Institute of New Jersey, Rutgers University, 08901 New Brunswick, NJ USA

**Keywords:** Cancer cell plasticity, EMP, cSCC, ITGAV, Prognostic biomarker, IGF1R

## Abstract

**Background:**

Early cutaneous squamous cell carcinomas (cSCCs) generally show epithelial differentiation features and good prognosis, whereas advanced cSCCs present mesenchymal traits associated with tumor relapse, metastasis, and poor survival. Currently, the mechanisms involved in cSCC progression are unclear, and the established markers are suboptimal for accurately predicting the clinical course of the disease.

**Methods:**

Using a mouse model of cSCC progression, expression microarray analysis, immunofluorescence and flow cytometry assays, we have identified a prognostic biomarker of tumor relapse, which has been evaluated in a cohort of cSCC patient samples. Phosphoproteomic analysis have revealed signaling pathways induced in epithelial plastic cancer cells that promote epithelial-mesenchymal plasticity (EMP) and tumor progression. These pathways have been validated by genetic and pharmacological inhibition assays.

**Results:**

We show that the emergence of epithelial cancer cells expressing integrin αV (ITGAV) promotes cSCC progression to a mesenchymal state. Consistently, ITGAV expression allows the identification of patients at risk of cSCC relapse above the currently employed clinical histopathological parameters. We also demonstrate that activation of insulin-like growth factor-1 receptor (IGF1R) pathway in epithelial cancer cells is necessary to induce EMP and mesenchymal state acquisition in response to tumor microenvironment-derived factors, while promoting ITGAV expression. Likewise, ITGAV knockdown in epithelial plastic cancer cells also blocks EMP acquisition, generating epithelial tumors.

**Conclusions:**

Our results demonstrate that ITGAV is a prognostic biomarker of relapse in cSCCs that would allow improved patient stratification. ITGAV also collaborates with IGF1R to induce EMP in epithelial cancer cells and promotes cSCC progression, revealing a potential therapeutic strategy to block the generation of advanced mesenchymal cSCCs.

**Graphical Abstract:**

During cSCC progression, cancer cells evolve from the epithelial to the mesenchymal state, which is associated with poor prognosis. The current investigation reveals that, at intermediate cSCC stages (MD/PD-SCC), epithelial cancer cells activate IGF1R and ITGAV signaling to acquire EMP and progress to the aggressive mesenchymal state in response to TME-derived factors. In addition, ITGAV allows the identification of these epithelial plastic cancer cells and functions as a prognostic biomarker of tumor relapse.

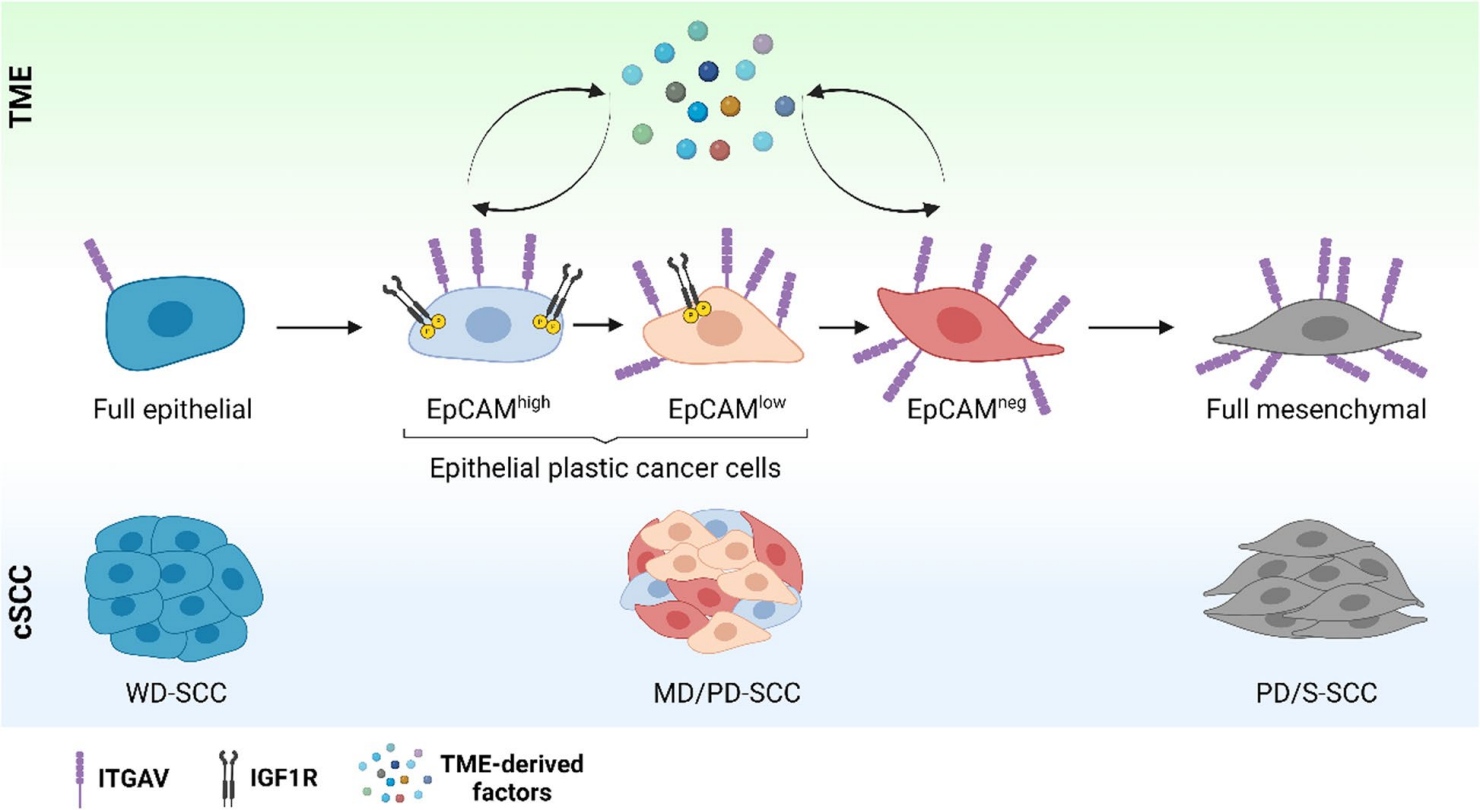

**Supplementary Information:**

The online version contains supplementary material available at 10.1186/s13046-024-03119-3.

## Background

The incidence of cutaneous squamous cell carcinoma (cSCC), the second most prevalent human skin cancer, is increasing worldwide [1, 2]. Although most cSCC cases are successfully removed by surgical excision, about 8% of patients suffer tumor relapse, which is associated with increased tumor aggressiveness, metastasis, and poor survival [3, 4]. Early cSCCs conserve epithelial differentiation traits and are considered well or moderately differentiated (WD/MD-SCCs, G1-G2 grade). However, advanced cSCCs exhibit poorly differentiated/spindle features (PD or PD/S-SCCs, G3-G4 grade), and have an enhanced risk of tumor relapse and metastasis [5, 6], and a limited response to radiotherapy, chemotherapy, and anti-PD-1 inhibitors [7, 8]. Therefore, it is essential to determine the mechanisms involved in the generation of advanced cSCCs to design new targeted therapies. In addition, since the current criteria based on the histopathological features of primary tumors and patient characteristics can be not efficient enough to determine aggressive progression and cSCC outcome [9], establishing prognostic biomarkers for tumor relapse is also an unmet need.

Our previous studies demonstrated that mouse epithelial WD-SCCs composed of epithelial cancer cells progressed to MD/PD-SCCs, and then to advanced PD/S-SCCs, which contained only mesenchymal cancer cells [10]. cSCC progression was associated with an expansion of the cancer stem cell (CSC) population and an induction of the epithelial-mesenchymal transition (EMT) program [10], which is involved in cancer cell plasticity, migration, and invasion [11]. The EMT program is a sequential process in which epithelial cancer cells switch to a mesenchymal state through intermediate/hybrid epithelial/mesenchymal (E/M) phenotypes [12]. Hybrid cancer cells retain epithelial features while acquiring mesenchymal ones, and show the greatest potential for phenotypic plasticity [13]. These hybrid E/M cells have been identified in several cancers, and are associated with enhanced metastasis and therapy resistance [14–17]. Nevertheless, whether the emergence of hybrid E/M cancer cells could be a risk factor for cSCC progression and tumor relapse is still largely unexplored. Unraveling the mechanisms involved in the acquisition of epithelial-mesenchymal plasticity (EMP) is key to blocking the generation of hybrid/mesenchymal cancer cells resistant to current therapies [18–21].

Here, we demonstrate that integrin αV (ITGAV) is a prognostic biomarker of tumor relapse, detecting cSCC epithelial plastic cancer cells with a hybrid E/M phenotype and an increased ability to progress to the aggressive mesenchymal phenotype. Identifying cSCC prognostic biomarkers that allow differentiation of epithelial plastic cancer cells from histopathologically identical epithelial non-plastic cancer cells is a key clinical step in the early identification of those patients at risk of developing tumor relapse. We further demonstrate that the activation of insulin-like growth factor-1 receptor (IGF1R) signaling pathway in epithelial cancer cells promotes ITGAV expression and induces EMP in response to tumor microenvironment (TME)-derived factors. Indeed, ITGAV contributes to IGF1R-mediated EMP in epithelial plastic cancer cells. However, IGF1R inhibition in mesenchymal cSCCs has no effect on cancer cell plasticity. These insights reveal therapeutic strategies to block the generation of advanced mesenchymal cSCCs and highlight the relevance of biomarkers in determining the optimal scenario for targeted therapies.

## Methods

### cSCC patient samples

cSCC patient samples were supplied by the Pathology Unit of the Hospital Universitario de Bellvitge and the HUB-ICO-IDIBELL Biobank (Barcelona, Spain). Age, gender, or ethnicity were not considered in the study design. The median age of patients in the cohort 1 at the time of primary cSCC surgery was 81 years, whereas in the cohort 2 it was 75.5 years.

### Mouse cSCC cancer cell cultures and in vitro treatment

Mouse cSCC cancer cells were derived from spontaneous or DMBA/TPA-induced tumors previously developed in K14-HPV16^Tg/+ ^mice [10]. Primary cSCC cancer cells isolated by fluorescence-activated cell sorting (FACS-sorting) from mouse WD-SCCs and MD/PD-SCCs were grown in basal DMEM-F12 medium (Life Technologies, 31331–093) supplemented with 1% penicillin/streptomycin (P/S, Biowest, L0022-100) and 1X B27 (Life Technologies, 17504–044). Cancer cells were cultured at 37ºC in a humidified 5% CO_2_ incubator. All cancer cells used in this study expressed green fluorescent protein (GFP) after transduction with an MSCV-IRES-GFP lentivirus plasmid.

Cancer cells were treated with 2.5 ng/μl TGFβ1 (dissolved in PBS-0.1% BSA, Peprotech, 100–21) for 14 days.

### Lentiviral transfection and cancer cell infection

To knockdown the expression of IGF1R and ITGAV, pLKO sh-control (Sigma, SHC002), sh-IGF1R (1) (TRCN0000023493), sh-IGF1R (2) (TRCN0000023490), sh-ITGAV (1) (TRCN0000066589), and sh-ITGAV (2) (TRCN0000066588) (Sigma, Mission® library) were used. Transfection was made in 293 T cells. Following a standard protocol, EpCAM^high^ and EpCAM^low^ cancer cells were infected with 293 T medium containing lentiviral particles. Transduced cancer cells were selected with 4 µg/ml of puromycin (Sigma, P8833). Knockdowns were confirmed by western blot.

### Animal studies and in vivo treatment

C57BL/6 and FVB mice colonies were maintained in the IDIBELL animal facility, whereas the athymic nude Foxn1^nu^ mice were purchased from Envigo. Mice were maintained in a temperature-controlled (23ºC), pathogen-free environment with a 12-h light/dark cycle, and with ad libitum access to food and water. All generated cSCCs were implanted into the back skin of 7–9-week-old male mice. Tumor volume (V = $\pi$/6 × L × W^2^; L: length, W: width) was measured every 2–3 days.

The frequency of tumor-initiating cells in WD-SCCs (OT14 lineage) was calculated beforehand using ELDA software [10] and 1,000 cancer cells (40-fold fewer than the estimated CSC frequency) were mixed 1:1 with Matrigel Basement Membrane matrix (Corning, 356234) and subcutaneously engrafted into the back skin of nude mice. When tumors reached a critical size, they were excised and small pieces were serially transplanted into a new nude mouse.

For the remaining in vivo studies, 1 × 10^4^ FACS-isolated GFP^+^ cancer cells were mixed 1:1 with Matrigel matrix and subcutaneously engrafted into the back skin of immunocompetent syngeneic mice (C57BL6/FVB F1 background). 3 × 10^3^ cancer cells were injected subcutaneously only in the comparison of GFP^+^EpCAM^+^ITGAV^+^ *vs.* GFP^+^EpCAM^+^ITGAV^−^ cancer cells. Tumors were excised when they reached critical size and processed for flow cytometry analysis.

OSI-906 (dissolved in 80% PBS + 13% PEG300 + 5% Tween-80 + 2% DMSO; MedChemExpress, HY-10191) in vivo treatment is detailed in the Supplementary Methods.

### Flow cytometry analysis and sorting

Excised mouse cSCCs were mechanically minced with a scalpel and enzymatically digested overnight at 37ºC with 1600 U/ml collagenase type I (Sigma, C0130) and 70 U/ml dispase (Life Technologies, 17105–041) in RPMI medium (Life Technologies, 61870044) supplemented with 10% fetal bovine serum (FBS, Life Technologies, 10270106), 20 mM HEPES (Sigma, H3537), and 1% antibiotic/antimycotic (Biowest, L0010-100). Cell suspensions were filtered and depleted of red blood cells with ammonium-chloride-potassium (ACK) lysis buffer (Lonza, BP10-548E) for 10 min at room temperature. To deplete endothelial cells, cell suspensions were incubated with a purified rat anti-mouse CD31 antibody (1:100, BD Bioscience, 550274) for 30 min at 4ºC with agitation, and then with Dynabeads anti-rat IgG (1:33, Life Technologies, 11035) for 30 min at 4ºC with agitation.

For flow cytometry analysis and FACS-sorting, 100,000 or 500,000 cells/condition, respectively, were incubated with 1 mg/ml IgG (Sigma, I5381) in blocking buffer (5% FBS in PBS) for 10 min at room temperature. Cells were then incubated for 30 min at 4ºC with the following antibodies: CD326 (EpCAM)-APC-eF780 1:400 (G8.8, eBioscience, 47–5791-82), CD45-APC 1:200 (30-F11, BioLegend, 103112), CD45-PE 1:300 (30-F11, TONBO, 50–0451-U100), CD49f-FITC 1:10 (GOH3, BD Biosciences, 555735), ITGAV (CD51)-PE 1:150 (RMV-7, BioLegend, 104106), ITGB3 (CD61)-APC 1:150 (2C9.G2, BioLegend, 104316), and VCAM1 (CD106)-APC-eF660 1:150 (429, eBioscience, 50–1061-82). Cells were washed with 0.5% BSA, 2 mM EDTA in PBS, and resuspended in analysis buffer (2% FBS, 2 mM EDTA in PBS).

Flow cytometry assays of in vitro-growing cancer cells started with the removal of adherent cells from culture plates with 2.5X Trypsin–EDTA (Life Technologies, 15400–054). Cancer cells were rested for 2 h in DMEM-F12 medium. 100,000 cells/condition were stained with anti-CD326 (EpCAM)-APC-eF780 1:600 (G8.8, eBioscience, 47–5791-82) or anti-ITGAV-PE 1:200 (RMV-7, BioLegend, 104106) antibodies in blocking buffer for 30 min at 4ºC. Cancer cells were then washed and resuspended in analysis buffer.

All flow cytometry assays were performed using BD FACSAria™ Fusion equipment. Live cells excluded 4’,6-diamidino-2-phenylindole (DAPI, Thermo Scientific, 62248). Data were analyzed with FlowJo v10.4.2 software.

### Tumor collection and histology

Small pieces of excised mouse cSCCs were fixed with 4% formaldehyde (PanReac, 252931) overnight and paraffin-embedded, or fixed for 30 min immediately after resection and embedded in optimal cutting temperature (OCT) medium to favor the visualization of GFP cancer cells. Patient paraffin-embedded cSCC sections were provided by the Pathology Unit and the HUB-ICO-IDIBELL Biobank (Barcelona, Spain).

### Immunofluorescence and immunohistochemistry assays

Cryosections from mouse cSCCs were defrosted at room temperature and washed for 5 min with Tris-buffered saline (TBS) at room temperature. Samples were then permeabilized in TBS-0.1% Triton X-100 (Sigma, 9036–19-5) for 15 min at room temperature, blocked with 5% horse serum (HS, Life Technologies, 16050122) in TBS for 1 h, and incubated overnight at 4ºC with the following primary antibodies: anti-CD326 (EpCAM) 1:50 (eBioscience, 14–5791-85), anti-ITGAV (CD51) 1:50 (Abcam, ab179475), anti-pIGF1R (Y1167/Y1168) 1:100 (biorbyt, orb14812), and anti-Vimentin 1:100 (Abcam, ab92547). Next, tumor sections were labelled for 1 h at room temperature with secondary antibodies: anti-rat Alexa Fluor 546 1:200 (Invitrogen, A-11081), anti-rat Alexa Fluor 647 1:100 (Invitrogen, A-48272), anti-rabbit Alexa Fluor 568 1:100 (Invitrogen, A-10042), and anti-rabbit Alexa Fluor 647 1:300 (Invitrogen, A-31573).

Patient paraffin-embedded cSCC sections were deparaffinized with xylol and rehydrated with decreasing alcohol concentrations. Antigen retrieval was performed with 10 mM TRIS/EDTA (pH 9.0), and samples were blocked with 5% HS in TBS for 1 h at room temperature. Samples were incubated overnight at 4ºC with primary antibodies: anti-E-cadherin 1:100 (BD Biosciences, 610182), anti-ITGAV (CD51) 1:50 (Abcam, ab16821), and anti-Vimentin 1:100 (Abcam, ab45939). The next day, tumor sections were incubated with secondary antibodies for 1 h at room temperature: anti-mouse Alexa Fluor 568 1:100 (Invitrogen, A-10037) or anti-rabbit Alexa Fluor 647 1:100 (Invitrogen, A-31573).

Nuclei were stained with DAPI 1:5000 (Invitrogen, D3571), and samples were mounted with Vectashield mounting medium (Palex, 416397). Images were captured with a 40X objective under a Leica TCS SP5 confocal microscope. Due to the great intratumor heterogeneity within cSCC patient samples, a representative number of images from different tumor areas was captured to obtain an accurate overview of each sample. Cancer cells were differentiated from the stroma cells by GFP expression or by nuclear atypia and cell size by training at the Pathology Service of the Hospital de Bellvitge. All images were analyzed using ImageJ v1.54d software.

pSMAD2 immunohistochemistry was performed in mouse paraffin-embedded samples. Antigen retrieval with 10 mM sodium citrate (pH 6.0) was performed in deparaffinized samples, followed by 3% H_2_O_2_ (Millipore, 1.07210.1000) incubation for 10 min. Samples were blocked with 5% HS in TBS and incubated overnight at 4ºC with anti-pSMAD2 1:500 (Cell Signaling, 3101). The next day, slides were incubated with a secondary anti-rabbit Envision System-HRP antibody (Dako, K4003), followed by DAB developing system (Dako, K3468), and counterstained with hematoxylin. Samples were visualized under light microscopy (Nikon Eclipse 80i).

### Quantitative real-time PCR (qRT-PCR) analysis

cDNA was obtained by Pico profiling in the Functional Genomics Core of the Institute for Research in Biomedicine (IRB, Barcelona, Spain) [22] or by High Capacity cDNA Reverse transcription kit (Applied Biosystems, 4374966) with RNA previously extracted with Trizol Reagent (Invitrogen, 15596026). qRT-PCR was carried out by mixing SYBR Green PCR Master Mix (Applied Biosystems, 4312704) with 4 µg of cDNA and the primers of interest listed in the Supplementary Methods. qRT-PCR reactions (3 replicates per sample) were performed on an Applied QuantStudio5 machine. All values were normalized relative to the expression of the *Gapdh* and *Ppia* housekeeping genes. The log_2_-fold change (FC) of mRNA levels was measured by relativizing the mean of mRNA levels with respect to the two housekeeping genes.

### Western blot

Total protein from cancer cells was extracted with lysis buffer containing 50 mM Tris pH 8.5 mM EDTA, 350 mM NaCl, 0.5% NP-40, 10% glycerol, 1 mM phenylmethylsulfonyl fluoride (PMSF), 2 mM NaF, 0.1 mM Na_3_VO_4_, 1 mM dithiothreitol (DTT), 1 × PhosSTOP™ (Roche, 04906845001), 1 × cOmplete™ (Roche, 11697498001), and 0.1% SDS (Invitrogen, 24730020). All samples underwent SDS-PAGE gel electrophoresis and then transferred to a nitrocellulose membrane (GE Healthcare, 10600001). Membranes were blocked with 5% BSA for at least 1 h at room temperature, followed by overnight incubation at 4ºC with the following primary antibodies: anti-AKT 1:1000 (Cell Signaling, 9272), anti-p-AKT 1:1000 (Cell Signaling, 9271), anti-ERK 1:1000 (Cell Signaling, 4696), anti-p-ERK 1:1000 (Cell Signaling, 4376), anti-IGF1R 1:1000 (Cell Signaling, 3027), anti-ITGAV 1:6000 (Abcam, ab179475), anti-p-SMAD3 (S423/425) 1:1000 (Abcam, ab52903), and anti-Actin-HRP 1:500,000 (Sigma, a3854). Membranes were incubated with anti-rabbit-HRP (Dako, P0448) or anti-mouse-HRP (Dako, P0260) secondary antibody for 1 h at room temperature, and protein-antibodies complexes were detected by chemiluminescence using ECL™ Western Blotting Detection Reagents (Cytiva, RPN2106).

### Microarray analysis

For the comparison of whole gene expression profiles of full epithelial cancer cells (α6-integrin^+^CD45^−^EpCAM^+^ cells isolated from 3 independent WD-SCCs) *vs.* EpCAM^+^ plastic cancer cells (α6-integrin^+^CD45^−^EpCAM^+^ cancer cells isolated from 3 independent MD/PD-SCCs), cDNA amplification by Pico profiling was performed as previously described [22]. cDNA was purified using column based PureLink Quick PCR Purification kit (Invitrogen). 8 µg of cDNA were used for hybridization in an Affymetrix Mouse Genome 430 PM array strip. Expression microarray analysis is detailed in the Supplementary Methods.

### Phosphoproteomic analysis

Phosphoproteomic analyses were performed at the OncoProteomics Laboratory (OPL) of the Amsterdam University Medical Center, location VUmc (Amsterdam, The Netherlands), following their previous protocols [23]. Detailed information in the Supplementary Methods.

### Statistical analysis

Statistical tests and graphs were generated using GraphPad Prism v8.0 or R v4.0.5. Statistical tests were selected according to the experimental setup, as detailed in the figure legends. Significant differences between tumor growths were analyzed by Repeated Measures ANOVA test. The association between ITGAV variable and tumor relapse was illustrated by plotting the relapse variable and the percentage of ITGAV^+^ cancer cells, and then fitting the smooth curve obtained by logistic regression analysis. The best cut-off point for the prognostic biomarker in cSCC patient samples and their diagnostic accuracy measures were obtained according to Youden’s index maximization criterion (ThresholdROC R package). Logistic regression models were used to establish the association between classic histopathological parameters and tumor relapse. Results were reported as odds ratios and 95% confidence intervals. Graphs illustrate means ± standard deviations (SDs); and sample sizes (*n*) are specified in the figure legends. Levels of statistical significance were designated as *P < 0.05, **P < 0.01, ***P < 0.001, ****P < 0.0001.

## Results

### Hybrid E/M phenotype identifies epithelial plastic cancer cells in mouse cSCCs

Our previous studies showed that cancer cell features change during cSCC progression [10, 24]. To characterize these dynamic changes and establish a model of tumor progression, we engrafted 40-fold fewer cancer cells than the estimated CSC frequency in our mouse WD-SCC lineages [10], reducing CSC heterogeneity. We generated WD-SCCs composed mainly of epithelial α6-integrin^+^EpCAM^+^ cancer cells from a putative epithelial-like CSC (Fig. S1A). The serial engraftment of these WD-SCCs into new immunodeficient mice gave rise to MD/PD-SCCs formed of epithelial α6-integrin^+^EpCAM^+^ and mesenchymal α6-integrin^+^EpCAM^−^ cancer cells (Fig. S1A). PD/S-SCCs containing only mesenchymal α6-integrin^+^EpCAM^−^ cancer cells were generated after MD/PD-SCC engraftment (Fig. S1A). Therefore, epithelial cancer cells acquire EMP, which promotes their progression to the mesenchymal state.

**Fig. 1:**
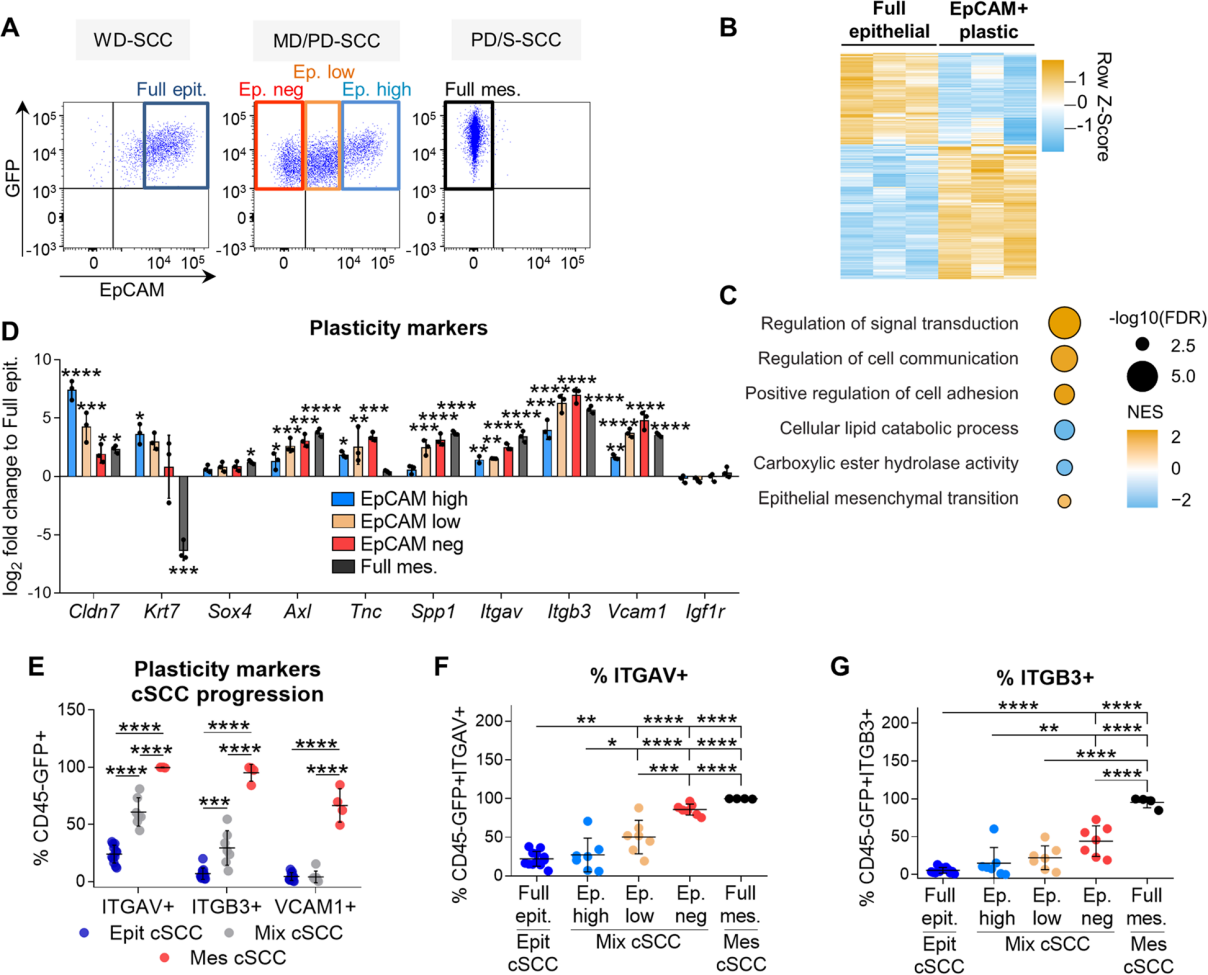
Identification of plasticity markers in mouse cSCCs. **A** Cancer cell populations isolated during mouse cSCC progression based on EpCAM expression: full epithelial (Full epit., GFP^+^CD45^−^EpCAM^high^) from WD-SCCs; EpCAM^high^ (Ep. high, GFP^+^CD45^−^EpCAM^high^), EpCAM^low^ (Ep. low, GFP^+^CD45^−^EpCAM^low^) and EpCAM^neg^ (Ep. neg, GFP^+^CD45^−^EpCAM^−^) from MD/PD-SCCs; and full mesenchymal (Full mes., GFP^+^CD45^−^EpCAM^−^) from PD/S-SCCs. **B** Euclidean hierarchical clustering of differentially expressed genes (log_2_-FC ≥ 1; FDR *P* < 0.05) between full epithelial (WD-SCCs) and EpCAM^+^ plastic (MD/PD-SCCs) cancer cells (*n* = 3/group). **C** Gene set enrichment analysis of EpCAM^+^ plastic *vs.* full epithelial cancer cells. The color scale represents the normalized enrichment score (NES), and the size of the bubbles the -log_10_ of the FDR. The signatures selected for this plot belong to Hallmark and Gene Ontology; FDR < 0.05 and NES > 0 for all cell types. **D** mRNA expression levels (mean ± SD) of plasticity genes in the indicated cancer cells relative to full epithelial cancer cells (*n* = 3/group). *P*-value (one-way ANOVA with Dunnett’s test). **E** Percentage (mean ± SD) of ITGAV^+^, ITGB3^+^, and VCAM1^+^ cancer cells in epithelial (*n* = 12), mixed (*n* = 7), and mesenchymal (*n* = 4) cSCCs. *P*-value (one-way ANOVA with Tukey’s test). **F**, **G** Percentage (mean ± SD) of (**F**) ITGAV^+^ and (**G**) ITGB3^+^ cells in the indicated cSCC cancer cells. *P*-value (one-way ANOVA with Tukey’s test)

For tracing, we transduced cancer cells with GFP. Cancer cells from WD-SCCs showed a high level of EpCAM expression and were designated full epithelial cancer cells, whereas epithelial EpCAM^+^ cancer cells from MD/PD-SCCs exhibited variable EpCAM expression and were classified as EpCAM^high^ or EpCAM^low^. In contrast, mesenchymal EpCAM^neg^ (from MD/PD-SCCs) and full mesenchymal (from PD/S-SCCs) cancer cells lost EpCAM expression (Fig. 1A). Molecular characterization of these cancer cell populations revealed that epithelial EpCAM^high^ and EpCAM^low^ cancer cells show dual expression of epithelial and mesenchymal genes, displaying a hybrid E/M phenotype that is not present in full epithelial cancer cells [21]. The engraftment of full epithelial cancer cells into immunocompetent syngeneic mice gave rise to tumors mostly composed of epithelial cancer cells. In contrast, EpCAM^high^ cancer cells generated tumors with a considerable percentage of EpCAM^neg^ cancer cells, whereas EpCAM^low^-derived tumors contained mostly mesenchymal cancer cells, demonstrating their increasing in vivo plasticity [21].

To determine the in vitro EMP of different epithelial cancer cell populations, we isolated by FACS-sorting and then cultured full epithelial, EpCAM^high^ and EpCAM^low^ cancer cells. During the first weeks, EpCAM^high^ and EpCAM^low^ cancer cells generated different epithelial and mesenchymal cancer cells, but only EpCAM^low^ cancer cells conserved a strong plasticity to reverse into EpCAM^high^ or to progress to the mesenchymal state after long-term in vitro culture (Fig. S1B). Full epithelial cancer cells never changed their phenotype (Fig. S1B).

The preferential acquisition of mesenchymal traits during cSCC growth indicates the involvement of TME-derived factors in forcing the shift towards a mesenchymal state in epithelial plastic cancer cells (EpCAM^high^ and EpCAM^low^). To further characterize the EMP of epithelial cancer cells under TME signals, we analyzed the in vitro response of full epithelial and epithelial plastic cancer cells to TGFβ, a factor involved in the induction of EMT in different cancer cell types [25]. Although in all cases the TGFβ1 treatment induced SMAD3 phosphorylation, confirming the activation of this pathway (Fig. S1C-S1E), the treatment did not promote EMP in full epithelial cancer cells (Fig. S1F). In contrast, EpCAM^high^ and EpCAM^low^ cancer cells gave rise to a higher percentage of mesenchymal cancer cells upon TGFβ1 treatment (Fig. S1G and S1H), indicating that only epithelial plastic cancer cells respond to TME-derived signals to promote cSCC progression. Furthermore, we observed that plastic EpCAM^high^ and EpCAM^low^ cancer cells did not induce the expression of *Tgfb1* and *Tgfb2* compared with full epithelial cancer cells (Fig. S1I), ruling out autocrine activation of the TGFβ pathway in epithelial plastic cancer cells to acquire the mesenchymal state. However, since epithelial plastic cancer cells can progress to the mesenchymal state in vitro without TGFβ (Fig. S1B), other intrinsic cellular factors must be involved in EMP.

Given the hybrid E/M phenotype exhibited by mouse cSCC plastic cancer cells, we evaluated their presence in different cSCC stages by analyzing the co-expression of epithelial EpCAM and mesenchymal Vimentin (Vim) markers in immunofluorescence (IF) assays (Fig. S2A). According to flow cytometry analyses, mouse tumors were classified as epithelial (WD-SCCs, < 30% EpCAM^neg^ cancer cells), mixed (MD/PD-SCCs, 30–90% EpCAM^neg^ cancer cells), or mesenchymal cSCCs (PD/S-SCCs, > 90% EpCAM^neg^ cancer cells). Epithelial GFP^+^EpCAM^+^Vim^−^ cancer cells made up the main cancer cell population in epithelial cSCCs and their frequency decreased during cSCC progression (Fig. S2B). In contrast, mesenchymal GFP^+^EpCAM^−^Vim^+^ cancer cells were most frequently detected in mesenchymal cSCCs, whereas Vim expression was associated with stromal cells in epithelial cSCCs (Fig. S2A and S2C). Finally, hybrid GFP^+^EpCAM^+^Vim^+^ cancer cells increased in mixed cSCCs (Fig. S2D), which are enriched in epithelial plastic cancer cells. These findings indicate the link between the emergence of hybrid E/M cancer cells and the promotion of cSCC progression.

Taken together, epithelial cancer cells are a heterogeneous population with different EMT degrees. EpCAM^high^ and EpCAM^low^ cancer cells present a hybrid E/M phenotype and strong plasticity for generating mesenchymal-aggressive cancer cells in contrast to full epithelial cancer cells. However, plastic and non-plastic epithelial cancer cells exhibit similar histopathological features that preclude their discrimination by current clinical criteria. Given the ability of hybrid E/M cancer cells to progress to the mesenchymal state, previously associated with aggressive cSCC growth and enhanced metastasis in mice [10], there is a need to identify epithelial plastic cancer cells, as well as the mechanisms involved in EMP acquisition.

### ITGAV is a specific marker of epithelial plastic cancer cells

To further identify molecular markers of epithelial plastic cancer cells, we compared the whole gene expression profile of full epithelial (from mouse WD-SCCs) and EpCAM^+^ plastic cancer cells (comprising EpCAM^high^ and EpCAM^low^ cancer cells from mouse MD/PD-SCCs). Expression microarray analysis revealed a set of 468 upregulated genes and 225 downregulated genes in EpCAM^+^ plastic cancer cells compared with full epithelial cancer cells (Fig. 1B; Additional file 2). Most of these altered genes were related to the upregulation of signal transduction, cell communication, and EMT (Fig. 1C). Specifically, our results showed an upregulation of *Cldn7*, *Krt7, Axl*, *Tnc*, *Itgav*, *Itgb3*, and *Vcam1* in EpCAM^high^ and EpCAM^low^ cancer cells relative to levels in full epithelial cancer cells (Fig. 1D).

Next, we focused on the protein expression levels of *Itgav, Itgb3*, and *Vcam1*, as they exhibited a progressive increase during mouse cSCC progression and had previously been associated with different EMT transition states in EpCAM^−^ skin and breast cancer cells [26]. Flow cytometry analyses showed that the percentage of ITGAV^+^ and ITGB3^+^ cancer cells was significantly higher in mixed compared with epithelial cSCCs, and that most cancer cells in mesenchymal cSCCs were ITGAV^+^ and ITGB3^+^ (Fig. 1E). In contrast, VCAM1 cell surface expression was only detected in mesenchymal cSCCs (Fig. 1E), discarding this marker for identifying early epithelial plastic cancer cells. This lack of correlation between VCAM1 mRNA and protein expression levels could be a consequence of posttranscriptional regulation [27]. Interestingly, the percentage of ITGAV^+^ cancer cells initially increased within the EpCAM^low^ population of mixed cSCCs, this marker being highly expressed in the mesenchymal cancer cells (Fig. 1F). However, the ITGB3^+^ population only increased within mesenchymal EpCAM^neg^ and full mesenchymal cancer cells (Fig. 1G), suggesting that the enrichment of ITGB3^+^ cancer cells in mixed cSCCs is a consequence of the emergence of EpCAM^neg^ cancer cells in these tumors (Fig. 1E). Accordingly, ITGB3 marker is not suitable for differentiating full epithelial from epithelial plastic cancer cells.

**Fig. 2:**
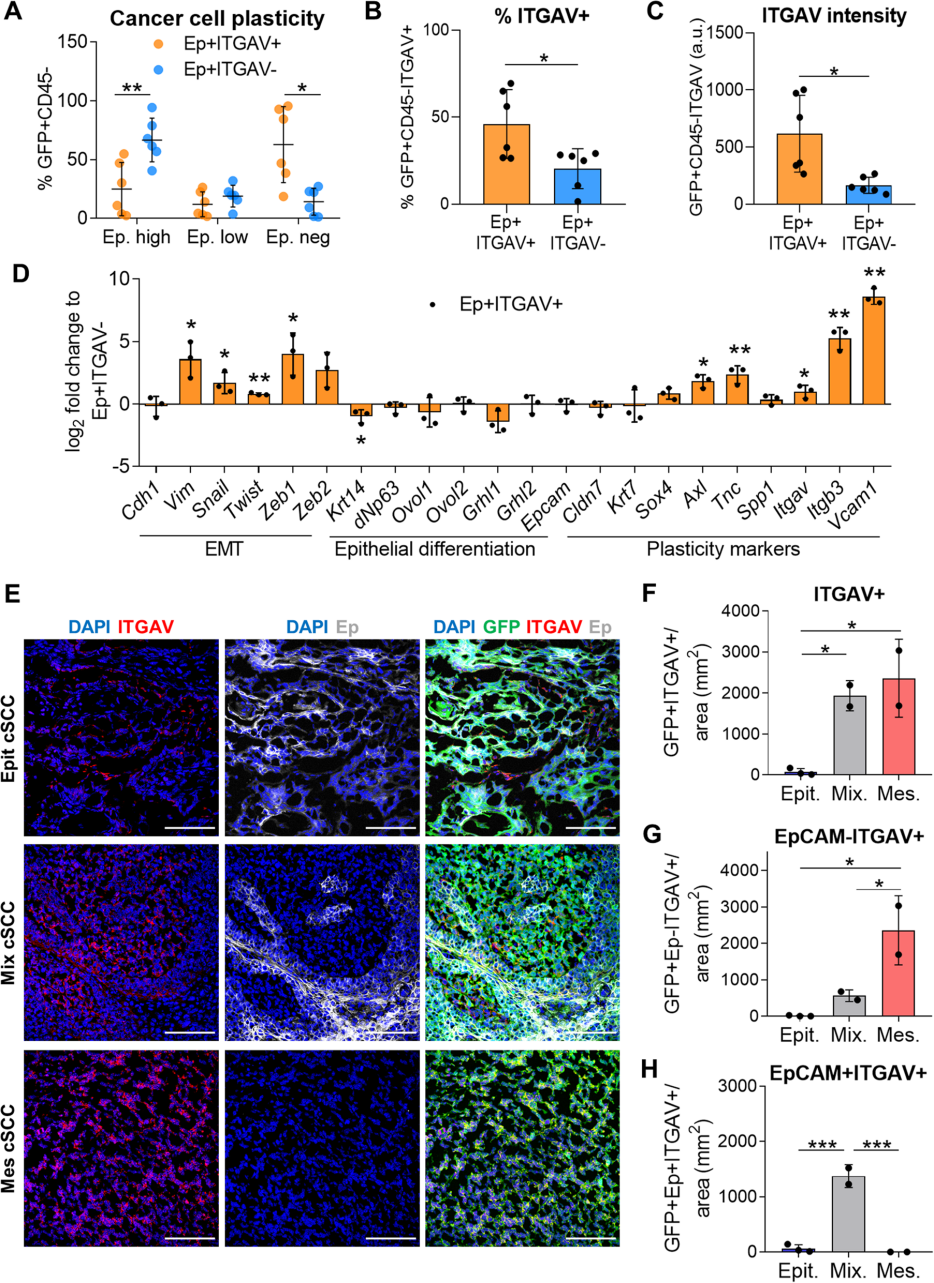
ITGAV marker identifies epithelial plastic cancer cells. **A** Percentage (mean ± SD) of EpCAM^high^, EpCAM^low^, and EpCAM^neg^ cancer cells generated after the engraftment of EpCAM^+^ITGAV^+^ and EpCAM^+^ITGAV^−^ cancer cells from mixed cSCCs into immunocompetent syngeneic mice (*n* = 6/group). *P*-value (unpaired two-tailed Student’s *t*-test). **B**, **C **(**B**) Percentage (mean ± SD) of ITGAV^+^ cancer cells and (**C**) median ITGAV intensity (mean ± SD) of cancer cells in EpCAM^+^ITGAV^+^ and EpCAM^+^ITGAV^−^-derived tumors. *P*-value (unpaired two-tailed Student’s *t*-test). **D** mRNA expression levels (mean ± SD) of the indicated genes in EpCAM^+^ITGAV^+^ cancer cells relative to EpCAM^+^ITGAV^−^ cancer cells from mixed cSCCs (*n* = 3/group). *P*-value (unpaired two-tailed Student’s *t*-test). **E** Representative IF images of GFP (green)/ITGAV (red)/EpCAM (Ep, white)-expressing cells in epithelial, mixed, and mesenchymal cSCCs. Nuclei labelled with DAPI (blue). Scale bar: 100 µM. **F**–**H** Quantification (mean ± SD) of (**F**) GFP^+^ITGAV^+^, (**G**) mesenchymal GFP^+^EpCAM^−^ITGAV^+^, and (**H**) epithelial GFP^+^EpCAM^+^ITGAV^+^ cancer cells per tumor area (mm^2^) in epithelial (*n* = 3), mixed (*n* = 2), and mesenchymal (*n* = 2) cSCCs. *P*-value (one-way ANOVA with Tukey’s test)

To test the plastic behavior of epithelial ITGAV^+^ cancer cells, we engrafted EpCAM^+^ITGAV^+^ and EpCAM^+^ITGAV^−^ cancer cells isolated from mixed cSCCs into immunocompetent syngeneic mice. EpCAM^+^ITGAV^+^ cancer cells gave rise to MD/PD-SCCs containing a higher percentage of mesenchymal EpCAM^neg^ and ITGAV^+^ cancer cells than EpCAM^+^ITGAV^−^-derived tumors (Fig. 2A-C). Indeed, EpCAM^+^ITGAV^−^ cancer cells gave rise to cSCCs similar to WD-SCCs, containing mostly EpCAM^high^ cancer cells (Fig. 2A). The molecular characterization of these EpCAM^+^ITGAV^+^ and EpCAM^+^ITGAV^−^ populations revealed that EpCAM^+^ITGAV^+^ cancer cells upregulated the expression of *Vim* and several EMT-transcription factors (EMT-TFs), while retaining the expression of most epithelial differentiation genes compared with EpCAM^+^ITGAV^−^ cancer cells (Fig. 2D). Likewise, EpCAM^+^ITGAV^+^ cancer cells expressed higher levels of the plasticity genes *Axl*, *Tnc*, *Itgb3*, and *Vcam1* than EpCAM^+^ITGAV^−^ cancer cells (Fig. 2D), indicating that EpCAM^+^ITGAV^+^ cancer cells exhibit a hybrid E/M phenotype. Therefore, ITGAV identifies within epithelial cancer cells those that start expressing mesenchymal genes and show a strong EMP potential to progress to the mesenchymal state.

Finally, we validated the potential of ITGAV to identify hybrid/plastic cancer cells by analyzing the expression of ITGAV and EpCAM in epithelial, mixed, and mesenchymal mouse cSCCs through standard clinical methods such as IF (Fig. 2E). We observed a significant increase in the frequency of ITGAV^+^ cancer cells in mixed cSCCs, and an even higher level in mesenchymal cSCCs (Fig. 2F). ITGAV expression was mainly observed in EpCAM^−^ cancer cells in mesenchymal cSCCs (Fig. 2G). In contrast, most of the ITGAV^+^ cancer cells in mixed cSCCs retained EpCAM expression (Fig. 2H). These results imply that ITGAV is a marker of epithelial plastic cancer cells.

### ITGAV is a prognostic biomarker for patient cSCC relapse

**Fig. 3:**
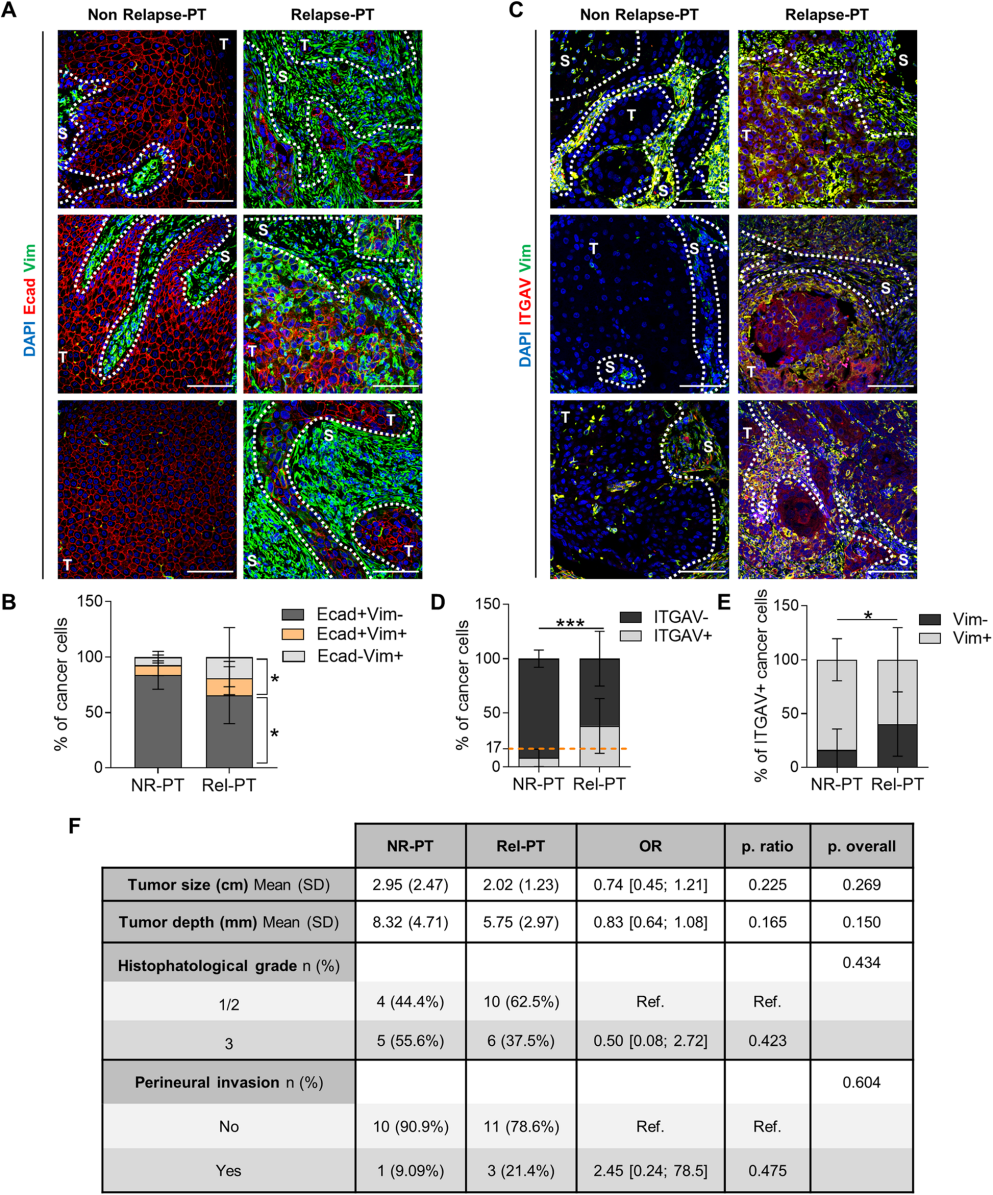
ITGAV is a prognostic biomarker of relapse in cSCC patients beyond current clinical parameters. **A** and **C** Representative IF images of (**A**) Ecad (red)/ Vim (green) and (**C**) ITGAV (red)/Vim (green)-expressing cells in 3 different NR-PT and Rel-PT samples. Nuclei labelled with DAPI (blue). Scale bar: 100 µM. T: tumor region; S: stroma region. **B** and **D** Percentage (mean ± SD) of (**B**) Ecad^+^Vim^−^, Ecad^+^Vim^+^, and Ecad^−^Vim^+^ and (**D**) ITGAV^−^ and ITGAV^+^ cancer cells in NR-PT (*n* = 11) and Rel-PT (*n* = 16) samples. Dotted orange line: percentage of ITGAV^+^ cancer cells above which it could be estimated as a risk factor for cSCC relapse. *P*-value (unpaired two-tailed Student’s *t*-test). **E** Percentage (mean ± SD) of Vim^−^ and Vim^+^ cancer cells within ITGAV^+^ cancer cell population in NR-PT (*n* = 11) and Rel-PT (*n* = 16) samples. *P*-value (unpaired two-tailed Student’s *t*-test). **F** Comparison of histopathological features of NR-PT and Rel-PT samples. OR: Odds ratio. Ref: reference group for OR. P. ratio: significance of the OR. P. overall: probability of the comparison of means (tumor size and depth) or proportions (histopathological grade and perineural invasion). *P*-values (unpaired two-tailed Student’s *t*-test for tumor size and depth, and Fisher’s exact test for histopathological grade and perineural invasion)

Given the aggressive growth and enhanced metastasis associated with patient cSCC relapses [3, 4] and the scarcity of prognostic biomarkers, we tested the plasticity marker ITGAV as a prognostic biomarker of tumor relapse in cSCC patient samples. We selected a cohort of cSCC patients who suffered local tumor relapse (Rel) within 18 months after surgical resection and cSCC patients who did not develop relapse (NR) during this period (Table 1). We first compared the expression of epithelial E-cadherin (Ecad) and mesenchymal Vim markers in the primary tumors of NR (NR-PT) and Rel (Rel-PT) patients by IF assays (Fig. 3A). NR-PT were highly enriched in epithelial Ecad^+^Vim^−^ cancer cells, whereas hybrid Ecad^+^Vim^+^ and mesenchymal Ecad^−^Vim^+^ cancer cells increased in Rel-PT samples (Fig. 3B and S3A). These findings suggest that hybrid E/M and mesenchymal cancer cells may be involved in cSCC relapse, as recently shown in breast cancer metastasis [28].

**Table 1:** Clinical information of cSCC patient samples: Relapse vs Non-relapse

	Patient	Histopathological features of primary tumors						
		Tumor size (maximum diameter) (cm)	Tumor depth (mm)	Histopathological grade	Perineural invasion	Surgical margins	Number of relapses	Time to first relapse (months)
Relapsed patients	Rel1	3.9	8	3	Yes	Clean	1	3
	Rel2	0.6	3.5	3	No	Clean	1	9
	Rel3	1.3	4	2	No	Clean	1	18
	Rel4	2.6	-	2	No	Clean	2	2
	Rel5	1.5	-	2	No	Clean	1	13
	Rel6	2.4	2	1	No	Clean	3	6
	Rel7	2.2	-	2	No	Clean	2	2
	Rel8	2.8	7	3	Yes	Clean	3	3
	Rel9	3.1	10	2	No	Clean	1	18
	Rel10	0.3	2	2	No	-	1	13
	Rel11	2.8	9	2	No	Clean	2	14
	Rel12	3.5	-	3	-	Focal invasion	1	0 (20 days)
	Rel13	3.5	8	2	Yes	Clean	3	8
	Rel14	0.5	-	3	-	Clean	1	15
	Rel15	0.6	4	3	No	Clean	1	6
	Rel16	0.7	-	1	No	Clean	4	13
Non-relapsed patients	NR1	0.7	4	-	No	Clean	0	-
	NR2	1.7	5	3	No	Clean	0	-
	NR3	2.7	6.5	2	Yes	Clean	0	-
	NR4	3	14	2	No	Clean	0	-
	NR5	1.5	4	2	No	Clean	0	-
	NR6	3.7	8	3	No	Clean	0	-
	NR7	1	7	3	No	Clean	0	-
	NR8	0.4	3	-	No	Clean	0	-
	NR9	4.9	9	3	No	Clean	0	-
	NR10	3.8	17	2	No	Clean	0	-
	NR11	9	14	3	No	Clean	0	-

Summary of the main histopathological features of primary tumors of 16 cSCC patients who relapsed (Rel) within 18 months after surgical resection and 11 cSCC patients who did not relapse (NR) during this period. Number of relapses and time to first relapse (months) are shown

This enrichment of hybrid/mesenchymal cancer cells in Rel-PT correlated with a higher percentage of ITGAV^+^ cancer cells in Rel-PT than in NR-PT samples (Fig. 3C and D). According to Youden’s index maximization criterion, the presence of more than 17% of ITGAV^+^ cancer cells in primary patient cSCCs is a risk factor for cSCC relapse in our cohort (Fig. 3D, S3C, and S3D). Most of the ITGAV^+^ cancer cells in NR-PT expressed Vim (Fig. 3E and S3B), indicating that ITGAV identifies the small Vim^+^ cancer cell population of these tumors. Interestingly, Rel-PT had a higher percentage of ITGAV^+^ cancer cells without Vim expression than NR-PT (Fig. 3E and S3B), suggesting that ITGAV also identifies an emerging epithelial plastic cancer cell population in Rel-PT samples that cannot yet be identified by mesenchymal markers in regular IF assays. Therefore, ITGAV is a prognostic biomarker of relapse for cSCC patients, since it is an early detector of cancer cell plasticity.

Currently, no molecular prognostic biomarkers of cSCC relapse have been established and the clinical risk factors for cSCC relapse are based on histological features of primary tumors, such as tumor size and depth, histopathological grade, and perineural invasion [3, 29]. However, we found that these histopathological factors were not significantly associated with cSCC relapse in our cSCC cohort (Fig. 3F), highlighting the prognostic value of ITGAV beyond the current clinical parameters. Altogether, our findings demonstrate that cSCC relapse is associated with an increased presence of ITGAV^+^ cancer cells in primary tumors.

### Patient cSCC relapses and metastases are enriched in Vim^+^ and ITGAV^+^ cancer cells

**Fig. 4:**
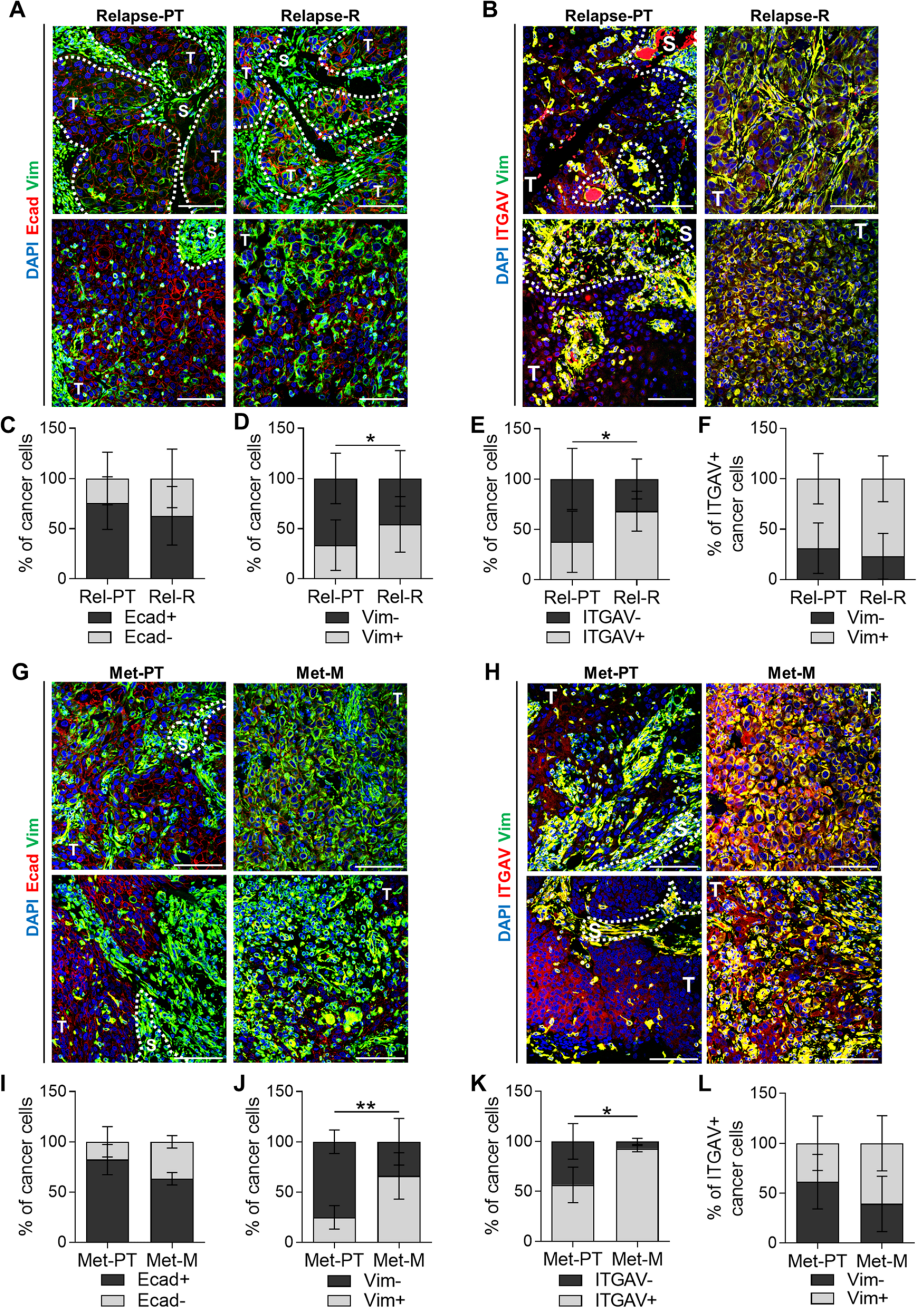
Patient cSCC local relapses and metastases have a higher frequency of Vim^+^ and ITGAV^+^ cancer cells than their respective primary tumors. **A**, **B** Representative IF images of (**A**) Ecad (red)/Vim (green) and (**B**) ITGAV (red)/Vim (green)-expressing cells in 2 different Rel-PT and Rel-R samples. Nuclei labelled with DAPI (blue). Scale bar: 100 µM. Dotted white line indicates T: tumor region; S: stroma region. **C**, **D** Percentage (mean ± SD) of (**C**) Ecad^−^ and Ecad^+^ and (**D**) Vim^−^ and Vim^+^ cancer cells in Rel-PT and Rel-R samples (*n* = 14/group). *P*-value (paired two-tailed Student’s *t*-test). **E** Percentage (mean ± SD) of ITGAV^−^ and ITGAV^+^ cancer cells in Rel-PT and Rel-R samples (*n* = 9/group). *P*-value (paired two-tailed Student’s *t*-test). **F** Percentage (mean ± SD) of Vim^−^ and Vim^+^ cancer cells within ITGAV^+^ population in Rel-PT and Rel-R samples (*n* = 9/group). *P*-value (paired two-tailed Student’s *t*-test). **G**, **H** Representative IF images of (**G**) Ecad (red)/Vim (green) and (**H**) ITGAV (red)/Vim (green)-expressing cells in 2 different Met-PT and Met-M samples. Nuclei labelled with DAPI (blue). Scale bar: 100 µM. Dotted white line indicates T: tumor region; S: stroma region. **I**-**K** Percentage (mean ± SD) of (**I**) Ecad^−^ and Ecad^+^, (**J**) Vim^−^ and Vim^+^, and (**K**) ITGAV^−^ and ITGAV^+^ cancer cells in Met-PT and Met-M samples (*n* = 4/group).* P*-value (paired two-tailed Student’s *t*-test). **L** Percentage (mean ± SD) of Vim^−^ and Vim^+^ cancer cells within ITGAV^+^ population in Met-PT and Met-M samples (*n* = 4/group). *P*-value (paired two-tailed Student’s *t*-test)

Since an increased frequency of hybrid/mesenchymal and ITGAV^+^ cancer cells was observed in Rel-PT samples, we analyzed whether, after relapse, these tumors were enriched in mesenchymal features associated with poor prognosis [10]. We compared the expression of Ecad, Vim, and ITGAV in cancer cells of primary tumors (Rel-PT) and in their respective local tumor relapses (Rel-R) (Table 1) using IF assays (Fig. 4A and B). Rel-R samples showed a slightly lower percentage of epithelial Ecad^+^ and a significant expansion of the mesenchymal Vim^+^ cancer cell population relative to Rel-PT samples (Fig. 4C and D), indicating a switch towards the mesenchymal cancer cell state during patient cSCC relapse. We noted significantly more ITGAV^+^ cancer cells in Rel-R compared with Rel-PT samples (Fig. 4E). Although we detected some ITGAV^+^ cancer cells without Vim expression in both conditions, ITGAV expression was closely associated with Vim expression (Fig. 4F), as previously observed in mouse cSCCs.

We also recruited a second cohort of patients who suffered distant metastases (Table 2). This cohort includes a small number of samples because the standard clinical practice is not to perform unnecessary biopsies or resections of metastatic cSCC lesions in elderly patients (median age of our cohorts between 75.5–81 years old) [30]. We compared the expression of Ecad, Vim, and ITGAV in cancer cells of primary tumors (Met-PT) and in their respective metastases (Met-M) (Fig. 4G and H). Consistent with the results observed in tumor relapses, Met-M samples slightly reduced the percentage of Ecad^+^ cancer cells (Fig. 4I) and were enriched in Vim^+^ (Fig. 4J) and ITGAV^+^ (Fig. 4K) cancer cells compared with Met-PT samples, with both conditions showing a similar percentage of ITGAV^+^Vim^+^ cancer cells (Fig. 4L). These findings point to an enrichment towards the mesenchymal phenotype after tumor relapse/metastasis in cSCC patient samples, reinforcing the need for prognostic biomarkers, such as ITGAV, to detect at early stages patients at risk of developing tumor recurrences.

**Table 2:** Clinical information of cSCC patient samples with metastases

	Patient	Histopathological features of primary tumors				
		Tumor size (maximum diameter) (cm)	Tumor depth (cm)	Histopathological grade	Perineural invasion	Surgical margins
Metastatic patients	Met1	3.4	2.2	3	No	Clean
	Met2	2	Dermis and subcutaneous infiltration	-	-	Clean
	Met3	8	2	2	No	Clean
	Met4	2.8	0.9	2	No	Clean

Main histopathological features of primary tumors of 4 cSCC patients who metastasized (Met)

### IGF1R signaling promotes EMP in epithelial cSCC cancer cells

**Fig. 5:**
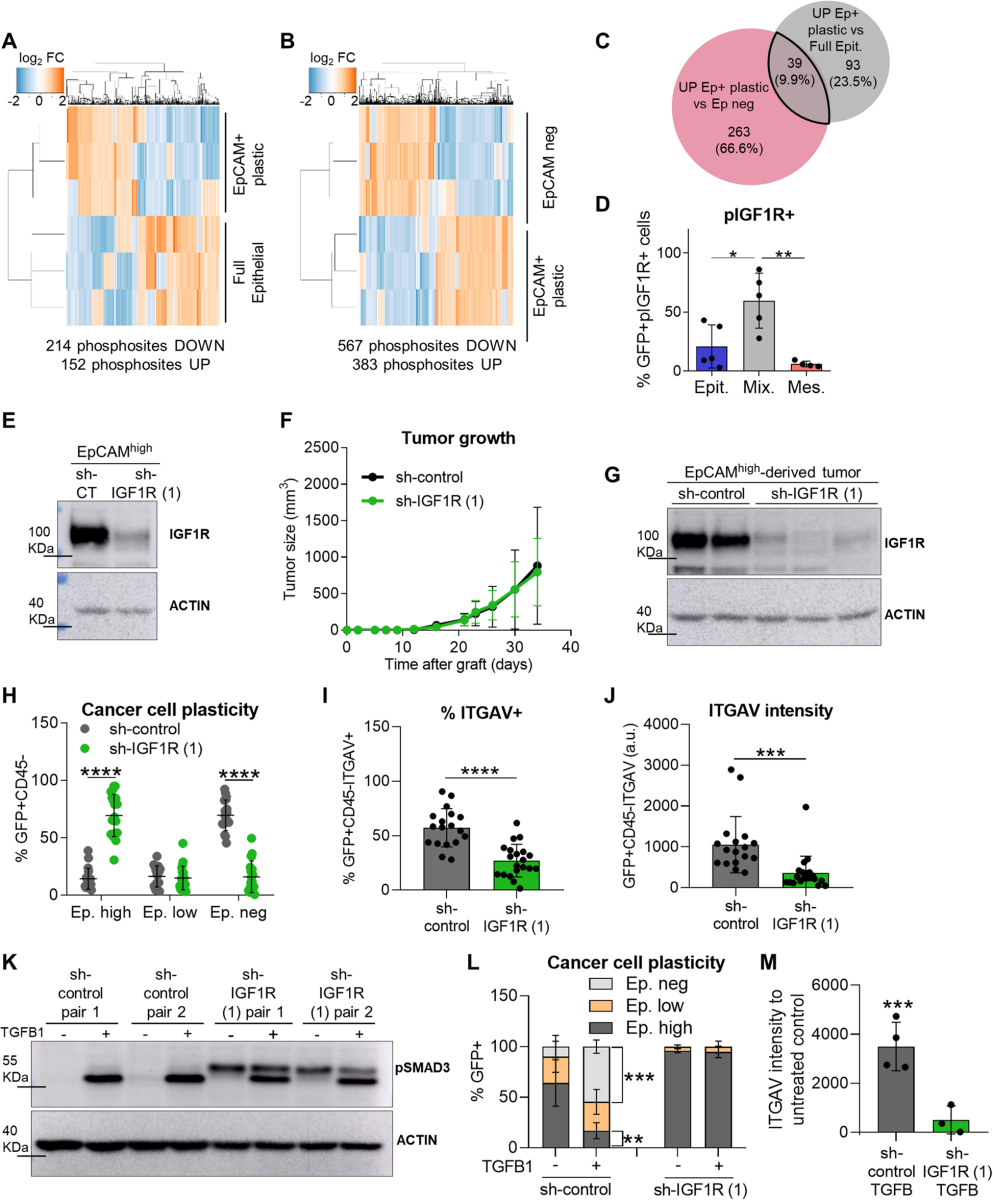
pIGF1R promotes EMP acquisition in epithelial cSCC cancer cells. **A**, **B** Hierarchical cluster analysis of differentially phosphorylated peptides (FDR *p* < 0.05) (**A**) between full epithelial and EpCAM^+^ plastic cancer cells isolated from WD-SCCs and MD/PD-SCCs, respectively, and (**B**) between EpCAM^+^ plastic and EpCAM^neg^ cancer cells isolated from MD/PD-SCCs (*n* = 3/group). **C** Venn diagram of the number of proteins specifically phosphorylated in the EpCAM^+^ plastic signature compared with full epithelial and EpCAM^neg^ cancer cells. **D** Percentage (mean ± SD) of GFP^+^pIGF1R^+^ cancer cells in epithelial (*n* = 5), mixed (*n* = 5), and mesenchymal (*n* = 4) cSCCs, determined by IF assays.* P*-value (one-way ANOVA with Tukey’s test). **E** Western blot confirmation of IGF1R knockdown in EpCAM^high^ sh-IGF1R (1) cancer cells prior to engraftment into immunocompetent syngeneic mice. Actin was used as a loading control. **F** Growth kinetics (mean ± SD) of EpCAM^high^ sh-control and sh-IGF1R (1)-derived tumors (n ≥ 18/group). *P*-value (Repeated Measures ANOVA test). **G** Representative image of IGF1R levels in the indicated cancer cells after tumor growth. Actin was used as a loading control. **H** Percentage (mean ± SD) of EpCAM^high^, EpCAM^low^, and EpCAM^neg^ cancer cells generated after the engraftment of EpCAM^high^ sh-control (*n* = 18) and sh-IGF1R (1) (*n* = 20) cancer cells into immunocompetent syngeneic mice. *P*-value (unpaired two-tailed Student’s *t*-test). **I**, **J **(**I**) Percentage (mean ± SD) of ITGAV^+^ cancer cells and (**J**) median ITGAV intensity (mean ± SD) of cancer cells in EpCAM^high^ sh-control and sh-IGF1R (1)-derived tumors. *P*-value (unpaired two-tailed Student’s *t*-test). **K** pSMAD3 levels after TGFβ1 (2.5 ng/µl) treatment for 14 days in the indicated cancer cells. Actin was used as a loading control. **L** Percentage (mean ± SD) of EpCAM^high^, EpCAM^low^, and EpCAM^neg^ cancer cells generated in TGFβ1-treated (+) and untreated (-) EpCAM^+^ plastic sh-control (*n* = 4) and sh-IGF1R (1) (*n* = 3) cancer cells. *P*-value (unpaired two-tailed Student’s *t*-test). **M** Median ITGAV intensity (mean ± SD) of sh-control (*n* = 4) and sh-IGF1R (1) (*n* = 3) cancer cells treated with TGFβ1 relative to their respective untreated controls. *P*-value (unpaired two-tailed Student’s *t*-test)

Next, we focused on determining signaling pathways involved in the acquisition of EMP in cSCC cancer cells. Using our mouse model of cSCC progression, we compared the phosphoproteome profile of full epithelial, EpCAM^+^ plastic, and mesenchymal EpCAM^neg^ cancer cells by high throughput mass spectrometry analysis. These analyses identified 152 enriched phosphosites belonging to 132 unique proteins, and 214 downregulated phosphosites belonging to 158 unique proteins in EpCAM^+^ plastic compared with full epithelial cancer cells (Fig. 5A and C; Additional file 3). Further changes in phosphosite composition were observed between epithelial EpCAM^+^ plastic and mesenchymal EpCAM^neg^ cancer cells, showing 302 upregulated proteins in EpCAM^+^ plastic *vs.* EpCAM^neg^ cancer cells (Fig. 5B and C). To identify pathways potentially involved in EMP acquisition, we compared the proteins with phosphosites upregulated in EpCAM^+^ plastic *vs.* full epithelial cancer cells with those upregulated in EpCAM^+^ plastic cancer cells *vs.* EpCAM^neg^ cancer cells. 39 phosphoproteins significantly increased in epithelial plastic cancer cells (Fig. 5C; Additional file 3), including the phosphorylation of the catalytic site of IGF1R (residues Y1167 and Y1168), which is involved in various aspects of cancer biology as transformation, cell growth, or therapy resistance [31, 32].

We validated these results by analyzing pIGF1R^+^ (Y1167 and Y1168) cancer cells during mouse cSCC progression using IF assays (Fig. S4A). As expected from the phosphoproteomic analysis, the percentage of pIGF1R^+^ cancer cells was significantly increased in mixed cSCCs, containing EpCAM^+^ epithelial plastic cancer cells, whereas this percentage practically disappeared in mesenchymal EpCAM^neg^ cancer cells from mesenchymal cSCCs (Fig. 5D, S4B, and S4C). Although we did not detect changes in *Igf1r* expression levels (Fig. 1D), IGF1R becomes phosphorylated and activated in epithelial plastic cancer cells, suggesting the involvement of this pathway in the EMP acquisition.

To test this hypothesis, we inhibited the activity of IGF1R in EpCAM^high^ cancer cells by using 2 different sh-RNA constructs. Sh-IGF1R and sh-control cells expressing IGF1R were engrafted into immunocompetent syngeneic mice (Fig. 5E and S5A). We observed that tumor growth was not delayed upon IGF1R abrogation (Fig. 5F and S5B), despite cancer cells maintained IGF1R interference during tumor growth (Fig. 5G and S5C). The analysis of cancer cell features after cSCC growth showed that the generation of mesenchymal EpCAM^neg^ cancer cells was significantly reduced in sh-IGF1R-derived tumors compared with the control group. Indeed, a strong accumulation of EpCAM^high^ cancer cells was observed in the sh-IGF1R cSCCs, resembling WD-SCCs composed mostly of epithelial cancer cells (Fig. 5H and S5D). In addition, sh-IGF1R cSCCs showed a significant reduction in the expression of ITGAV and in the percentage of ITGAV^+^ cancer cells compared with sh-control ones (Fig. 5I-J and S5E-S5H), indicating a loss of plasticity following IGF1R abrogation. Accordingly, the abrogation of IGF1R signaling reduced the response of epithelial plastic cancer cells to TGFβ1, blocking the generation of mesenchymal cancer cells and the upregulation of EMT-TFs, even though canonical and non-canonical TGFβ signaling did not appear to be altered upon IGF1R inhibition (Fig. 5K-L, S5I-S5J, and S6A-C). TGFβ1 also induced ITGAV expression in sh-control plastic cancer cells, while this effect was significantly reduced in sh-IGF1R cancer cells (Fig. 5M and S5K). Thus, activation of IGF1R signaling promotes EMP and tumor progression to a mesenchymal state. Analysis of *Igf1* and *Igf2* expression levels showed that epithelial plastic cancer cells did not induce the expression of these factors (Fig. S5L), suggesting a paracrine activation of IGF1R. Furthermore, as no significant changes in EMT and epithelial differentiation genes were observed in EpCAM^high^ cancer cells after abrogating IGF1R signaling (Fig. S5M), our results indicate that the role of IGF1R in EMP acquisition is more complex than the direct regulation of EMT-TFs expression previously reported in other tumor types [33–35].

**Fig. 6:**
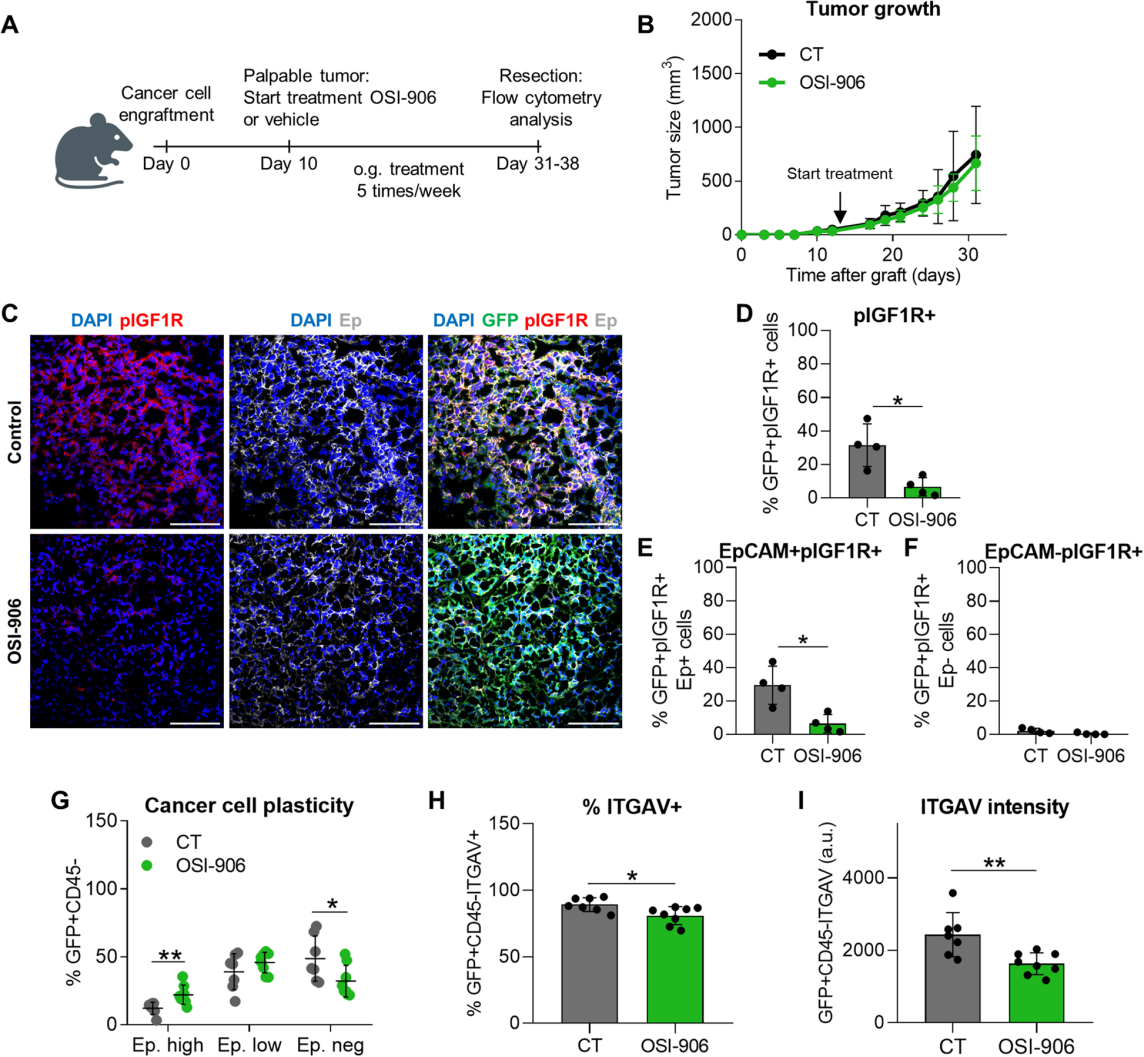
Pharmacological inhibition of IGF1R activity reduces EMP in mixed cSCCs despite not altering tumor growth. **A** Experimental design for the treatment of mixed cSCCs with vehicle (control) or OSI-906 (IGF1R inhibitor). When the grafted tumors were palpable, mice were treated orally 5 times per week with 30 mg/kg/dose. **B** Growth kinetics (mean ± SD) of control and OSI-906-treated mixed cSCCs (n ≥ 7/group). *P*-value (Repeated Measures ANOVA test). **C** Representative IF images of GFP (green)/pIGF1R (red)/Ep (white)-expressing cells in control and OSI-906-derived cSCCs. Nuclei stained with DAPI (blue). Scale bar: 100 µm. **D**-**F** Percentage (mean ± SD) of (**D**) total GFP^+^pIGF1R^+^, (**E**) epithelial GFP^+^EpCAM^+^pIGF1R^+^, and (**F**) mesenchymal GFP^+^EpCAM^−^pIGF1R^+^ cancer cells in control (*n* = 4) and OSI-906 (*n* = 4) mixed cSCCs. *P*-value (unpaired two-tailed Student’s *t*-test). **G** Percentage (mean ± SD) of EpCAM^high^, EpCAM^low^, and EpCAM^neg^ cancer cells generated in control (*n* = 7) and OSI-906 (*n* = 8) mixed cSCCs, as determined by flow cytometry analysis. *P*-value (unpaired two-tailed Student’s *t*-test). **H**, **I **(**H**) Percentage (mean ± SD) of ITGAV^+^ cancer cells and (**I**) median ITGAV intensity (mean ± SD) of cancer cells in control and OSI-906-derived tumors. *P*-value (unpaired two-tailed Student’s *t*-test)

To corroborate the involvement of IGF1R signaling in cancer cell plasticity acquisition, we pharmacologically inhibited IGF1R signaling in mixed cSCCs with OSI-906 inhibitor [36]. For this purpose, EpCAM^high^ cancer cells were engrafted into immunocompetent syngeneic mice, and when tumors were palpable, they were treated with OSI-906 or vehicle solution (Fig. 6A). OSI-906 treatment did not reduce tumor growth (Fig. 6B), despite blocking IGF1R signaling (Fig. 6C-F). However, pIGF1R inhibition significantly reduced the generation of mesenchymal cancer cells, as well as the percentage of cancer cells expressing ITGAV (Fig. 6G-I).

To evaluate at which cancer cell state IGF1R signaling is relevant for cSCC progression, we also abrogated IGF1R expression in EpCAM^low^ cancer cells using 2 different sh-RNA constructs (Fig. S7A). IGF1R interference in these epithelial plastic cancer cells did not affect the cSCC growth rate (Fig. S7B). In contrast to the results observed after IGF1R abrogation in EpCAM^high^ cancer cells, loss of IGF1R signaling in EpCAM^low^ cancer cells did not affect the ability of these hybrid cancer cells to generate mesenchymal cSCCs (Fig. S7C). No differences in the ITGAV marker were observed between EpCAM^low^ sh-IGF1R and sh-control-derived cSCCs (Fig. S7D and S7E).

In summary, activation of IGF1R signaling is necessary for the acquisition of cancer cell plasticity, priming epithelial plastic cancer cells for the paracrine action of TME-derived factors at early/intermediate stages of cSCC progression. At later stages, cancer cells can progress to the mesenchymal phenotype in response to stromal signals already independently of IGF1R signaling.

### Abrogation of ITGAV expression blocks the progression to the mesenchymal state of cSCC cancer cells

**Fig. 7:**
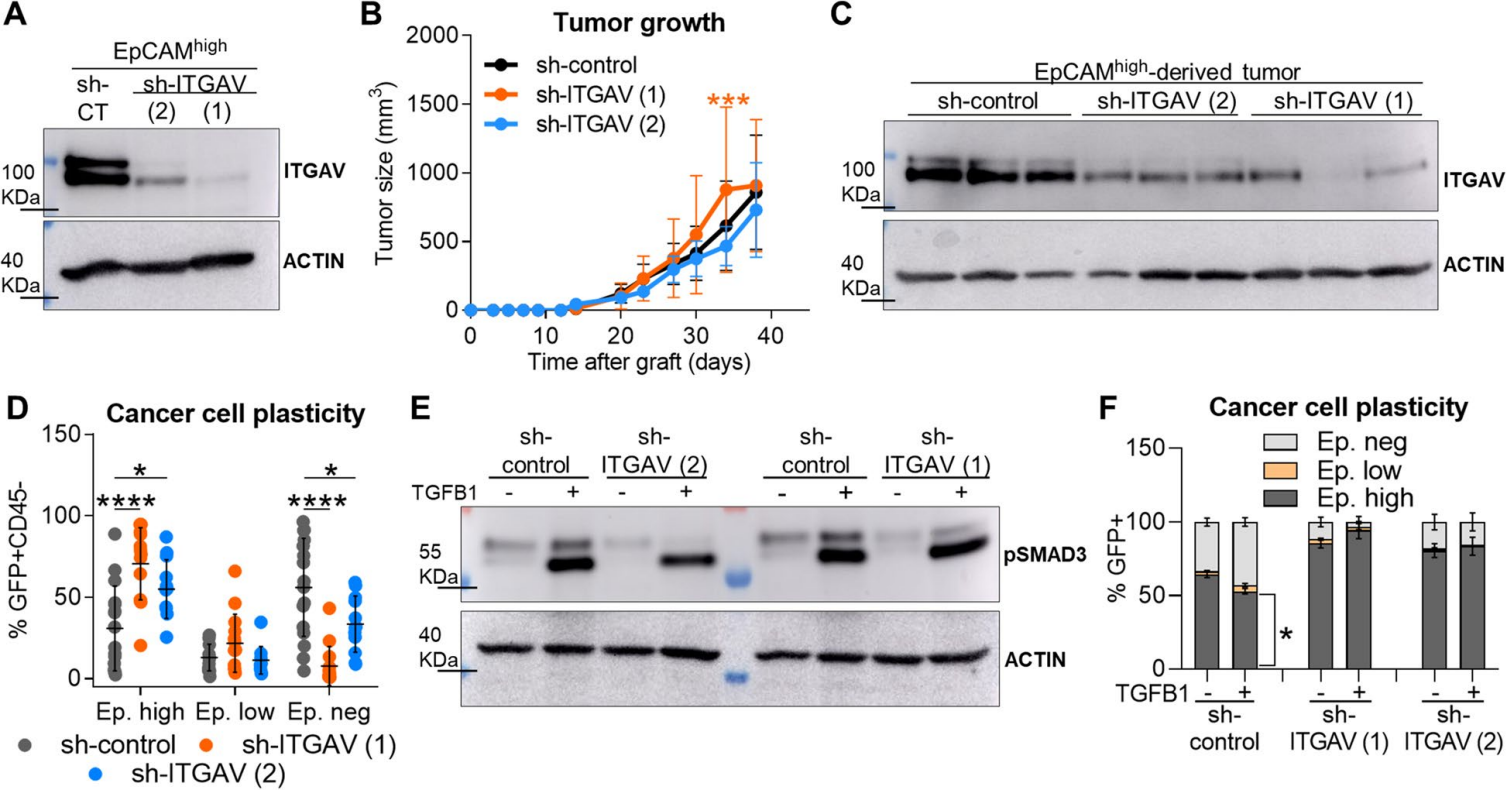
ITGAV abrogation in EpCAM^high^ cancer cells reduces the generation of mesenchymal cancer cells in derived tumors. **A** Demonstration of ITGAV abrogation in EpCAM^high^ sh-ITGAV (1 and 2) cancer cells compared with sh-control cancer cells prior to engraftment into immunocompetent syngeneic mice, determined by western blot. Actin was used as a loading control. **B** Growth kinetics (mean ± SD) of EpCAM^high^ sh-control and sh-ITGAV (1 and 2)-derived tumors (n ≥ 12/group). *P*-value (Repeated Measures ANOVA test). **C** Representative image of ITGAV expression in the indicated cancer cells after tumor growth. Actin was used as a loading control. **D** Percentage (mean ± SD) of EpCAM^high^, EpCAM^low^, and EpCAM^neg^ cancer cells generated after the engraftment of EpCAM^high^ sh-control (*n* = 16), sh-ITGAV (1) (*n* = 14), and sh-ITGAV (2) (*n* = 12) cancer cells into immunocompetent syngeneic mice. *P*-value (one-way ANOVA with Dunnett’s test). **E** Western blot image of pSMAD3 induction after TGFβ1 (2.5 ng/µl) treatment for 14 days in the indicated cancer cells. Actin was used as a loading control. **F** Percentage (mean ± SD) of EpCAM^high^, EpCAM^low^, and EpCAM^neg^ cancer cells generated in TGFβ1-treated (+) and untreated (-) EpCAM^+^ plastic sh-control (*n* = 2), sh-ITGAV (1) (*n* = 3), and sh-ITGAV (2) (*n* = 2) cancer cells. *P*-value (unpaired two-tailed Student’s *t*-test)

Since our results showed that activation of IGF1R signaling induces the expression of ITGAV, we analyzed whether ITGAV signaling may play a role in the acquisition of the mesenchymal phenotype. To this end, we interfered ITGAV expression in EpCAM^high^ cancer cells (Fig. 7A) and then engrafted sh-control and sh-ITGAV cancer cells into immunocompetent syngeneic mice. We observed that tumor growth was not delayed after ITGAV abrogation (Fig. 7B), despite cancer cells retained ITGAV down-regulation during tumor growth (Fig. 7C). The generation of mesenchymal EpCAM^neg^ cancer cells from epithelial plastic cancer cells was significantly reduced in sh-ITGAV-derived tumors (Fig. 7D), leading to a strong accumulation of EpCAM^high^ cancer cells, similarly to that observed in the sh-IGF1R cSCCs. Moreover, ITGAV knockdown in EpCAM^+^ plastic cancer cells significantly reduced EMT induction in response to TGFβ and blocked the generation of mesenchymal EpCAM^neg^ cancer cells, although cancer cells showed pSMAD3 induction upon TGFβ1 treatment and translocation of pSMAD2 to the nucleus (Fig. 7E-F and S8A-B). No changes in non-canonical TGFβ signaling were observed after ITGAV inhibition (Fig. S8C). Altogether, these results indicate that activation of IGF1R signaling promotes EMP and tumor progression toward a mesenchymal state in an ITGAV-mediated process, which facilitates the response to TME-derived factors, such as TGFβ.

## Discussion

Advanced PD/S-SCCs with mesenchymal features show aggressive growth and enhanced metastasis compared with early WD-SCCs [5, 6, 10]. In addition, advanced and metastatic cSCCs are usually more resistant to different therapies [7, 8]. Our previous studies demonstrated that mesenchymal PD/S-SCCs are generated by the malignant progression of epithelial WD-SCCs in mice [10]. At intermediate stages, MD/PD-SCCs show epithelial cancer cells that acquire EMP, which favors the progression of epithelial cancer cells to the aggressive mesenchymal state. These epithelial plastic cancer cells (identified as EpCAM^high^ and EpCAM^low^ cancer cells) exhibit a hybrid E/M phenotype characterized by the co-expression of epithelial and mesenchymal markers, in contrast to the full epithelial cancer cells from early WD-SCCs [21]. Here, we demonstrate the positive correlation between tumor relapse and the presence of hybrid/mesenchymal cancer cells in primary tumors of patient cSCC samples. We also found that patient cSCCs are enriched in hybrid and mesenchymal cancer cells after local tumor relapse and distant metastasis, showing the progression to a more aggressive mesenchymal stage in patients’ recurrences, similar to that reported in our mouse cSCC progression model.

As the emergence of hybrid E/M cancer cells is clinically relevant because it is also associated with metastasis and resistance to therapy in other tumor types [13, 14, 16, 17, 28, 37], we focused on identifying biomarkers of this hybrid population. Molecular characterization of mouse cSCC cancer cells allowed us to identify ITGAV as a marker of hybrid E/M cancer cells. In contrast to previous studies that focused on ITGAV expression in mesenchymal EpCAM^−^ cancer cells [26], our data show an increased ITGAV expression within epithelial EpCAM^+^ cancer cells that correlates with the emergence of epithelial plastic cancer cells. Furthermore, EpCAM^+^ITGAV^+^ cancer cells from mixed cSCCs exhibit a hybrid E/M phenotype and a higher ability to switch to the mesenchymal state than EpCAM^+^ITGAV^−^ cancer cells, demonstrating the value of ITGAV as a biomarker for identifying epithelial plastic cancer cells within a heterogeneous population of epithelial cancer cells. Although ITGAV expression is almost undetectable under physiological conditions, its increased expression has been observed in lung and pancreatic cancers [38, 39]. In fact, ITGAV was considered a marker of poor survival and progression in some tumors [40–43]. However, its prognostic value in cSCC progression has not been previously described. Here, we show that ITGAV expression in primary cSCC patient samples is predictive of tumor relapse, although it would be interesting to analyze this marker in an independent cohort. The translational relevance of our results lies in the role of the ITGAV marker to detect patients at risk of cSCC relapse by using a routine technique like IF. The identification of novel prognostic biomarkers complementing the information provided by established histopathological criteria will enable the accurate stratification of cSCC patients as part of progress towards personalized medicine.

The relevance of hybrid E/M cancer cells in tumor progression and relapse highlights the need to identify the mechanisms involved in their generation. Extracellular signals including Hepatocyte Growth Factor (HGF), Fibroblast Growth Factor (FGF), Platelet-Derived Growth Factor (PDGF), Epidermal Growth Factor (EGF), and pro-inflammatory cytokines, have been involved in EMT induction in solid carcinomas, although TGFβ1 is the most effective and characterized EMT initiator [44, 45]. The crosstalk of TGFβ with Wnt, NFkB, Notch or hypoxia signaling pathways orchestrates tumor-specific EMT progression through SMADS, SNAI1/2, TWIST, and ZEB1/2 factors [44, 46]. Our data show that cSCC cells are sensitive to TGFβ-induced EMT only at determined states of tumor progression. TGFβ-mediated EMT is only observed in epithelial plastic cancer cells, even though full epithelial cancer cells from WD-SCCs also exhibit a functional TGFβ signaling transduction upon TGFβ1 treatment. These results indicate that other signaling pathways activated in epithelial plastic cancer cells may contribute with TGFβ and/or other extracellular signals to induce the mesenchymal state, since not all epithelial cancer cells are prone to respond to EMT inducing signals [13, 47].

In this regard, we have identified IGF1R activation as a key driver of EMP acquisition in mouse cSCC epithelial cancer cells, leading to tumor progression and an aggressive mesenchymal state in response to TGFβ. IGF1/IGF1R pathway has been implicated in various aspects of cancer biology, such as cell transformation, EMT induction, invasion or metastasis, making it a desirable oncology therapeutic target [32, 35, 48]. IGF1R abrogation or inhibition in mixed cSCCs blocks epithelial plastic cancer cells in an epithelial state, reducing the generation of mesenchymal cancer cells. Contrary to the direct connection established between the IGF1R pathway and the regulation of EMT-TFs expression in other tumor types [33–35], we have not observed changes in EMT-TFs expression following IGF1R inhibition. These results indicate that the regulation of EMT by IGF1R in cSCCs is more complex than previously described, and other partners, such as ITGAV, could be involved. Similar to the IGF1R pathway, ITGAV signaling has been also linked to EMT, stemness or invasion, being ITGAV a critical activator of latent TGFβ [49–52]. We observed that IGF1R inhibition reduces ITGAV expression in cancer cells, accordingly with the EMP reduction of these cells. Previous studies have suggested an interaction between IGF1R and ITGAV in muscle cells, which require activation of the αVβ3 integrin to enhance migration promoted by IGF1R signaling [53, 54]. Interestingly, we show that ITGAV knockdown in EpCAM^high^ cancer cells generates epithelial cSCCs as those obtained after IGF1R inhibition, demonstrating the involvement of ITGAV in EMP. In addition, the lack of IGF1R or ITGAV renders epithelial cancer cells insensitive to TGFβ-mediated EMT induction, as demonstrated in lung cancer cells following IGF1R inhibition [55]. Similarly, TGFβ-induced ITGAV expression observed in epithelial plastic control cells, and previously described [56], is lost following IGF1R inhibition. However, TGFβ signaling is functional in the absence of IGF1R or ITGAV, as abrogation of these proteins did not affect phosphorylation and nuclear translocation of SMAD proteins, nor non-canonical TGFβ signaling assessed by pAKT and pERK levels [57]. We suggest that IGF1R and ITGAV promote EMP in response to TGFβ in cSCCs by mechanisms still unknown, although other TME-derived factors beyond TGFβ may contribute to EMT induction [16, 44, 58]. Therefore, our results indicate that activation of the IGF1R pathway is required for EMP acquisition and tumor progression to the aggressive mesenchymal state via ITGAV, priming epithelial cancer cells to TME-derived EMT-promoting signals, such as TGFβ.

Despite the necessity of the IGF1R pathway for initial EMP acquisition, the importance of this pathway is lost in advanced stages of cSCC progression. Indeed, EpCAM^low^ cancer cells with an already acquired cell plasticity, can evolve to the mesenchymal state independently of the IGF1R pathway, generating mesenchymal cSCCs. Over past years, IGF1R inhibitors have been developed with disappointing results in phase II/III trials in advanced and/or relapsed tumors [59–61]. No biomarkers of IGF1R activity were used in patient selection in these trials despite the preclinical evidence of their convenience [62, 63]. These failed trials may be due to therapy not being administered at the appropriate tumor stage, as our results demonstrate a narrow therapeutic window in which anti-IGF1R therapies are useful.

## Conclusions

Epithelial cancer cells at intermediate stages of mouse cSCC progression (MD/PD-SCCs) acquire EMP, exhibiting a hybrid E/M phenotype and an enhanced ability to generate mesenchymal-aggressive cancer cells. Our findings demonstrate that ITGAV expression identifies these epithelial plastic cancer cells, which cannot be discriminated from epithelial non-plastic cancer cells by current clinical criteria. Likewise, ITGAV expression allows early identification of those cSCC patients who are at risk of tumor relapse, reinforcing the need for prognostic biomarkers in cSCC patients’ management. In addition, we demonstrate that IGF1R signaling is required for EMP acquisition in response to TME-derived factors in an ITGAV-mediated process in cSCC epithelial cancer cells, whereas IGF1R inhibition in mesenchymal cSCCs has no effect on EMP. These insights reveal potential therapeutic strategies to block the generation of aggressive mesenchymal cSCCs and highlight the relevance of biomarkers in determining the window of opportunity for targeted therapies.

### Supplementary Information

Supplementary Material 1. This file includes Figures S1 to S8 and their respective figure legends, and Supplementary Methods.

Supplementary Material 2. Genes differentially expressed between full epithelial cancer cells and EpCAM^+^ plastic cancer cells.

Supplementary Material 3. Phosphoproteomic analysis of full epithelial, EpCAM^+^ plastic and EpCAM neg cancer cells.

## Data Availability

All data are provided with the article. The gene expression data described in Fig. 1B and Additional file 2 have been deposited in the Gene Expression Omnibus (GEO) database under accession number GSE233382. Phosphoproteomic analysis described in Fig. 5A-C and Additional file 3 have been deposited in ProteomXchange under accession number PXD048323. Any additional data used to support this work are available from the corresponding author upon request.

## Abbreviations

CSC: Cancer stem cell
cSCC: Cutaneous squamous cell carcinoma
E/M: Epithelial/mesenchymal
Ecad: E-cadherin
EMP: Epithelial-mesenchymal plasticity
EMT: Epithelial-mesenchymal transition
GFP: Green fluorescent protein
IF: Immunofluorescence
IGF1R: Insulin-like growth factor-1 receptor
ITGAV: Integrin αV
ITGB3: Integrin β3
MD: Moderately differentiated
NR: Non-relapsed patient
PD/S: Poorly differentiated/Spindle
Rel: Relapsed patient
R: Relapse
TGFβ: Transforming growth factor β
TME: Tumor microenvironment
VCAM1: Vascular cell adhesion molecule 1
Vim: Vimentin
WD: Well differentiated
