# Supplementary material for: IGF1R signaling induces epithelial-mesenchymal plasticity via ITGAV in cutaneous carcinoma
Source: J Exp Clin Cancer Res. 2024 Jul 29;43:211. doi: 10.1186/s13046-024-03119-3 (PMC11285232; doi:10.1186/s13046-024-03119-3)
Supplement: Supplementary file 1 — Supplementary Material 1. This file includes Figures S1 to S8 and their respective figure legends, and Supplementary Methods. [file 13046_2024_3119_MOESM1_ESM.pdf]

Supplementary Materials for:

IGF1R signaling induces epithelial-mesenchymal plasticity via ITGAV in cutaneous carcinoma

Marta Lopez-Cerda[1*], Laura Lorenzo-Sanz[1], Victoria da Silva-Diz[1,6], Sandra Llop[2], Rosa M. Penin[3], Josep Oriol Bermejo[4], Richard R de Goeij-de Haas[5], Sander R Piersma[5], Thang V Pham[5], Connie R. Jimenez[5], Juan Martin-Liberal[2*], Purificación Muñoz[1*]

* **Correspondence**: Marta Lopez-Cerda: mlopezc@idibell.cat, Juan Martin-Liberal: jmartinliberal@iconcologia.net, Purificación Muñoz: p.munoz@idibell.cat.

This PDF file includes:

Supplementary Figure S1-S8

Supplementary Methods

Supplementary References

SUPPLEMENTARY FIGURES

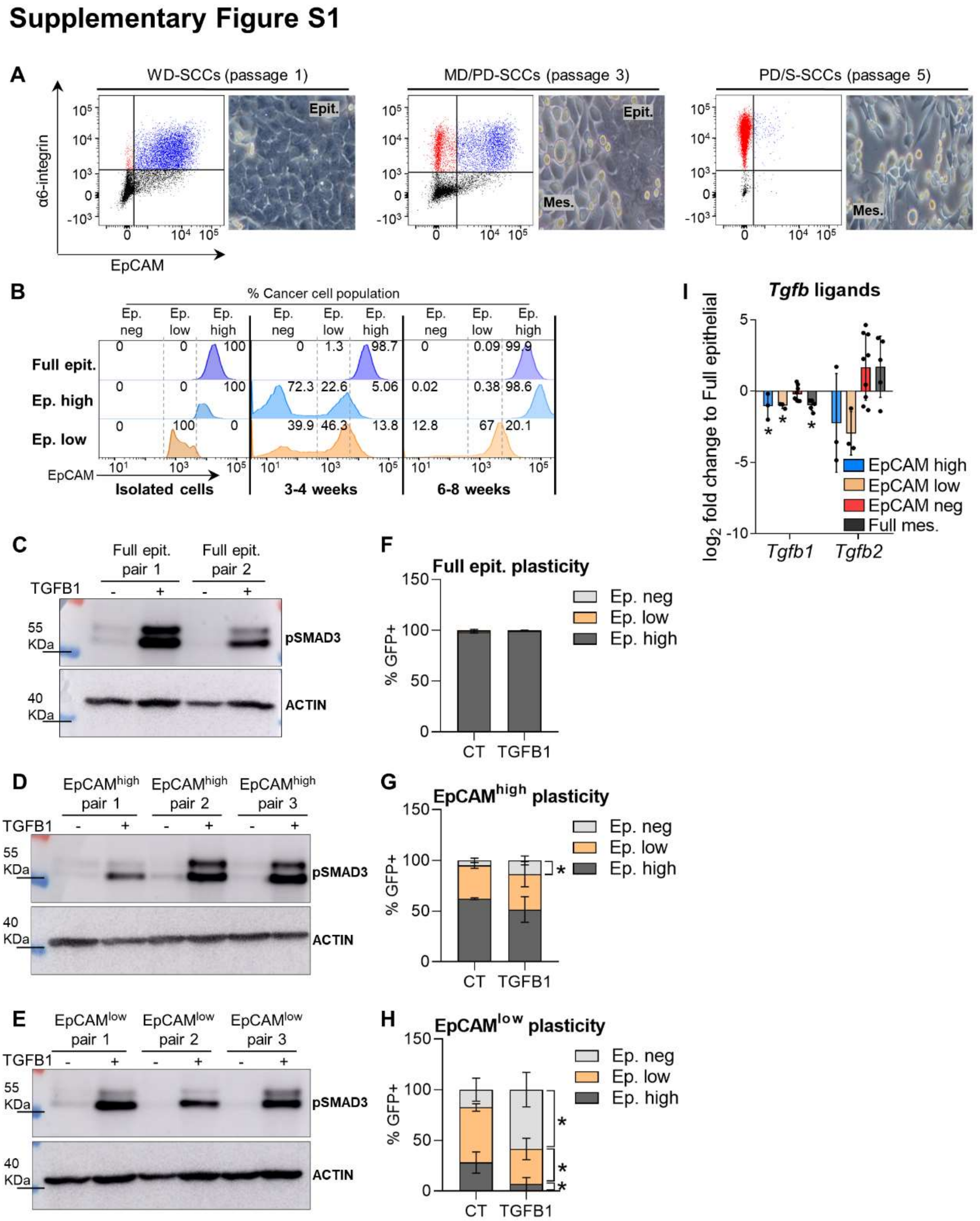

Supplementary Figure S1. Hybrid E/M cancer cells show strong plasticity for switching to the mesenchymal state.

A Representative flow cytometry plots showing the α6-integrin^{+}EpCAM^{+} and α6-integrin^{+}EpCAM^{-} cancer cell populations within WD-SCCs, and derived MD/PD-SCCs and PD/S-SCCs. Representative images of *in vitro*-growing cancer cells isolated from

WD-SCCs, MD/PD-SCCs and PD/S-SCCs, indicating the epithelial (Epit.) or mesenchymal (Mes.) phenotype. **B** Representative histograms showing the percentage of $EpCAM^{high}$, $EpCAM^{low}$, and $EpCAM^{neg}$ cancer cells generated under *in vitro* growth from isolated full epithelial, $EpCAM^{high}$, and $EpCAM^{low}$ cancer cells. Cancer cell plasticity was evaluated at 3-4 and 6-8 weeks after initial sorting. Before the 6-8 weeks analysis, cancer cells were sorted again to rescue the initial phenotype. **C-E** pSMAD3 levels in the indicated cancer cells after being untreated (-) or treated (+) with TGFβ1 2.5 ng/µl for 14 days. Actin was used as a loading control. **F-H** Percentage (mean ± SD) of $EpCAM^{high}$, $EpCAM^{low}$, and $EpCAM^{neg}$ cancer cells generated from TGFβ1-treated (+) and untreated (-) (**F**) full epithelial, (**G**) $EpCAM^{high}$, and (**H**) $EpCAM^{low}$ cancer cells (n = 3/group). *P*-value (unpaired two-tailed Student's *t*-test). **I** mRNA expression levels (mean ± SD) of *Tgfb1* and *Tgfb2* genes in the indicated cancer cells relative to full epithelial cancer cells (n ≥ 3/group). *P*-value (one-way ANOVA with Dunnett's test).

Supplementary Figure S2

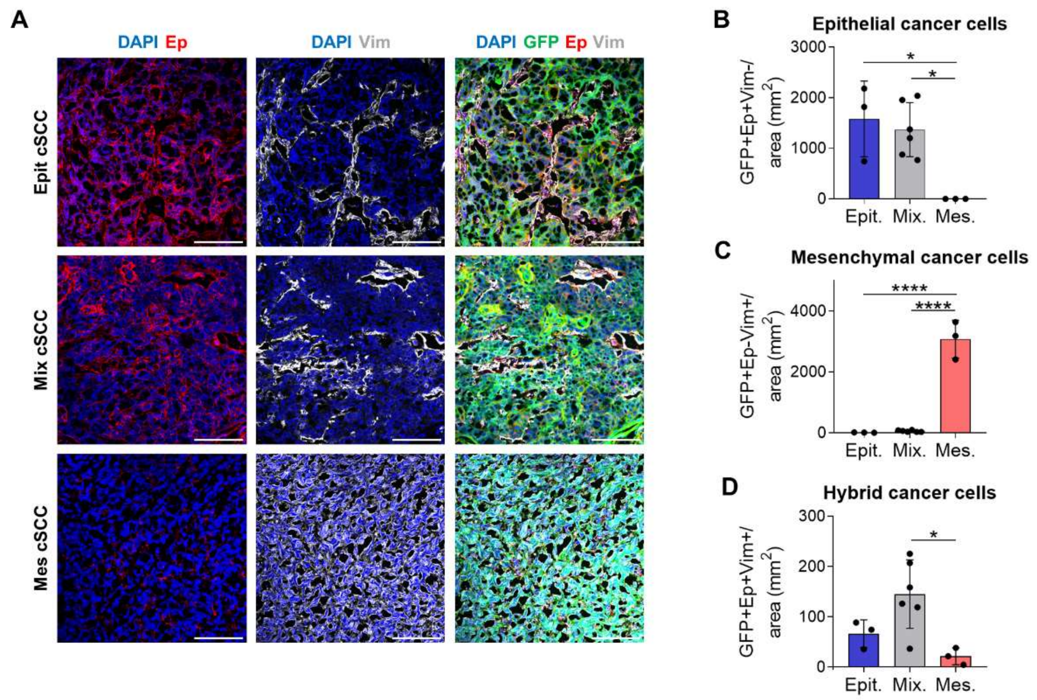

Supplementary Figure S2. Mixed cSCCs are enriched in hybrid E/M cancer cells.

A Representative IF images of GFP (green)/Ep (red)/Vim (white)-expressing cells in epithelial, mixed, and mesenchymal mouse cSCCs. Nuclei stained with DAPI (blue). Scale bar: 100 μm. **B**-**D** Quantification (mean ± SD) of (**B**) epithelial ($GFP^+Ep^+Vim^-$), (**C**) mesenchymal ($GFP^+Ep^-Vim^+$), and (**D**) hybrid ($GFP^+Ep^+Vim^+$) cancer cells per tumor area (mm^2) in epithelial (n = 3), mixed (n = 6), and mesenchymal (n = 3) cSCCs. *P*-value (one-way ANOVA with Tukey's test).

Supplementary Figure S3

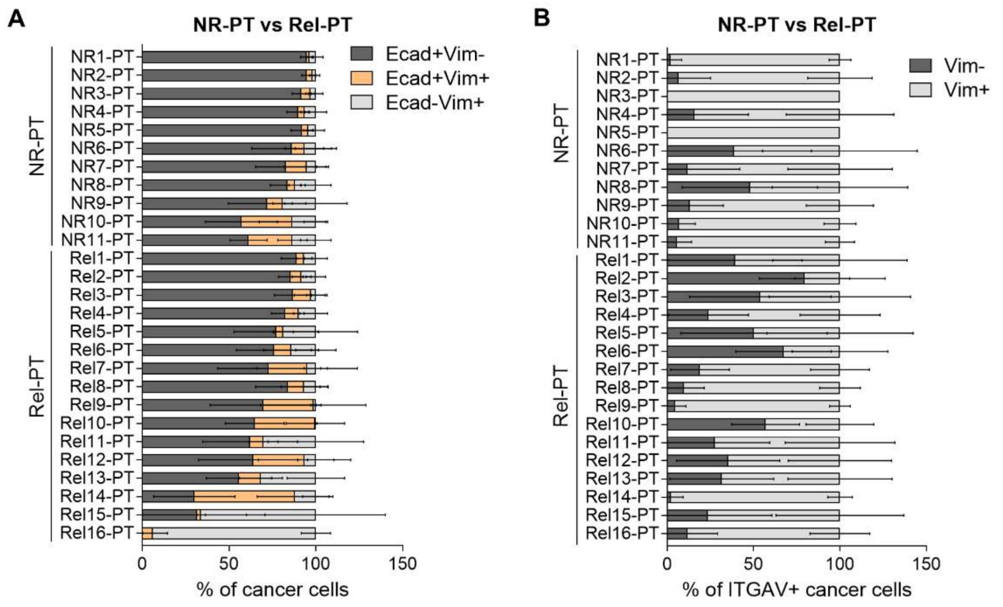

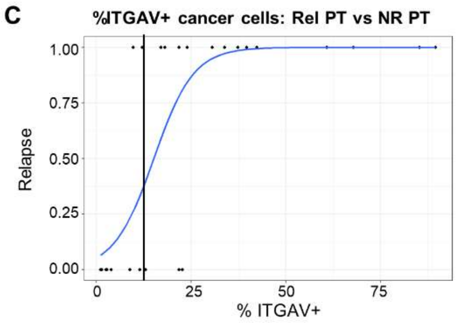

D

	Estim.	Low lim. (95%)	Up lim. (95%)
**Sensitivity**	0.875	0.617	0.984
**Specificity**	0.818	0.482	0.977
**Pos.Pred. Val.**	0.875	0.617	0.984
**Neg.Pred. Val.**	0.818	0.482	0.977

Supplementary Figure S3. Primary cSCCs with a higher percentage of ITGAV are at risk of relapsing.

A-B Percentage (mean ± SD) of (**A**) Ecad^{+}Vim^{-}, Ecad^{+}Vim^{+} and Ecad^{-}Vim^{+} cancer cells or (**B**) Vim^{+} and Vim^{-} cells within ITGAV^{+} cancer cells in the indicated cSCC patient samples. **C** Smoothed logistic regression curve with 95% confidence interval (CI) relating the percentage of ITGAV^{+} cancer cells and the probability of tumor relapse (0 = non relapse; 1 = relapse). **D** Diagnostic accuracy measures for the cut-off point obtained for the percentage of ITGAV^{+} cancer cells above which it could be estimated as a risk factor for cSCC relapse. Pos. Pred. Val.: positive predictive value; Neg. Pred. Val: negative predictive value.

Supplementary Figure S4

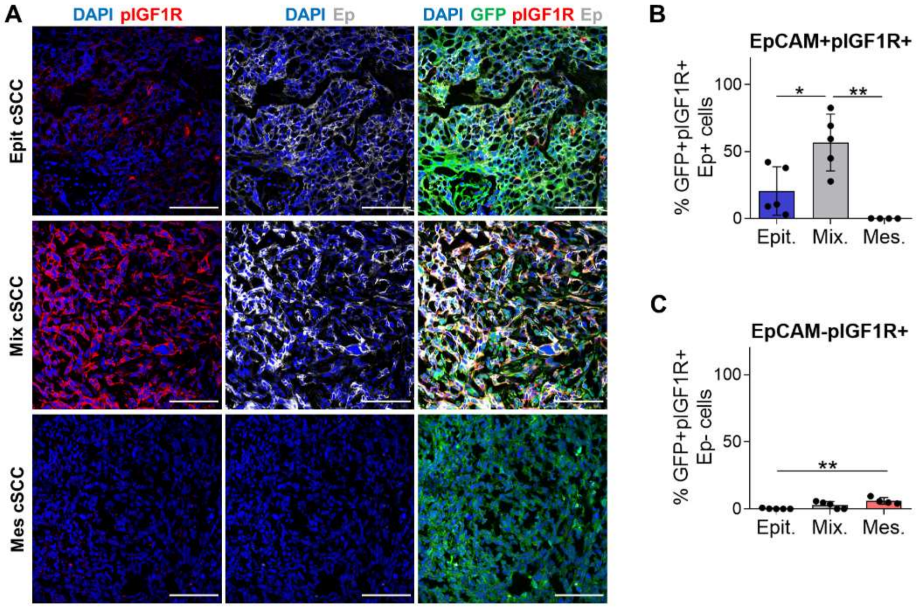

Supplementary Figure S4. Mixed cSCCs are enriched in EpCAM^{+}pIGF1R^{+} cancer cells.

A Representative IF images of GFP (green)/pIGF1R (red)/Ep (white)-expressing cells in epithelial, mixed, and mesenchymal mouse cSCCs. Nuclei stained with DAPI (blue). Scale bar: 100 µm. **B**-**C** Percentage (mean ± SD) of (**B**) epithelial GFP^{+}EpCAM^{+}pIGF1R^{+} and (**C**) mesenchymal GFP^{+}EpCAM^{-}pIGF1R^{+} cancer cells in epithelial (n = 5), mixed (n = 5), and mesenchymal (n = 4) cSCCs. *P*-value (one-way ANOVA with Tukey's test).

Supplementary Figure S5

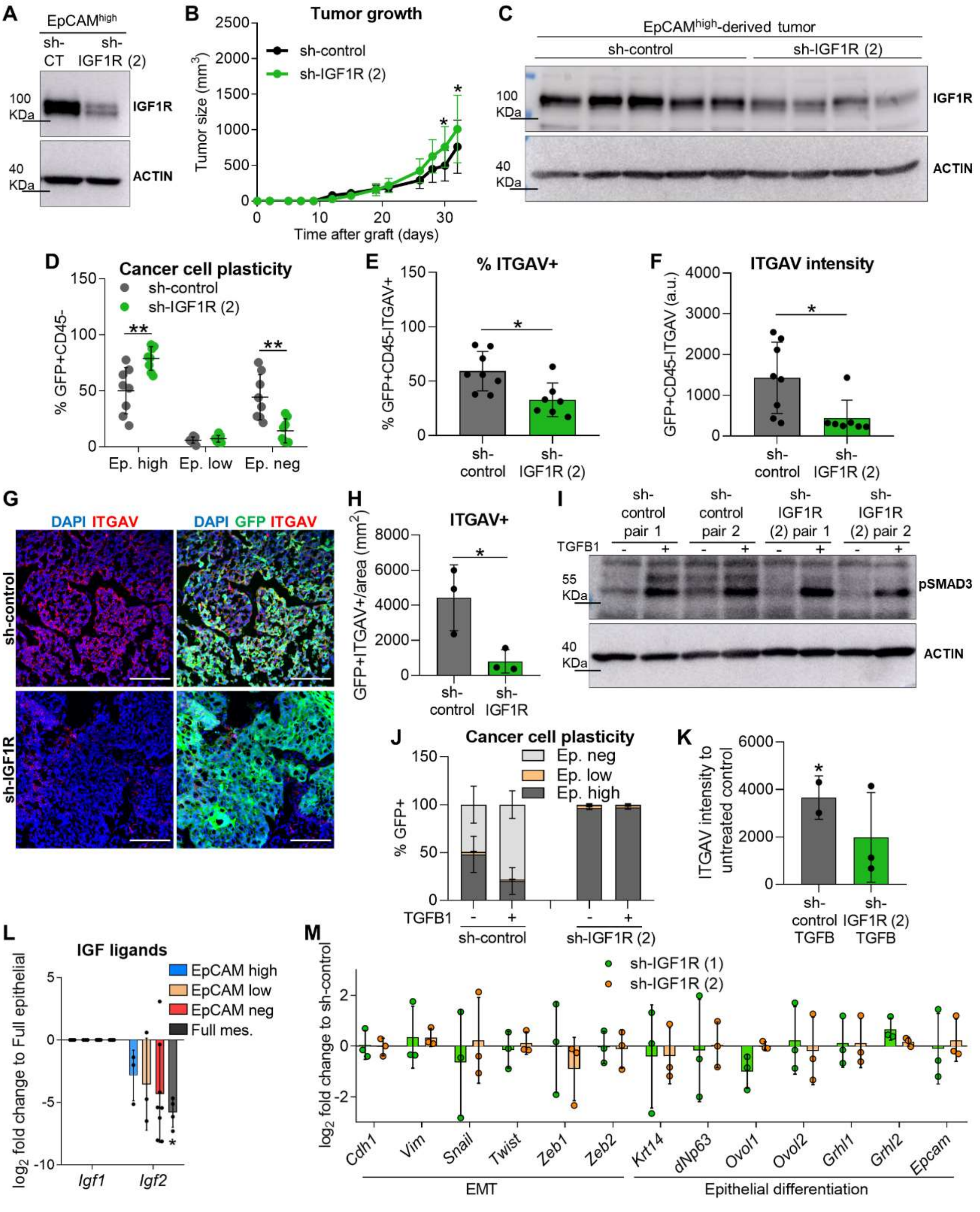

Supplementary Figure S5. IGF1R inhibition in EpCAM^{high} cancer cells blocks the generation of mesenchymal cancer cells and reduces ITGAV expression in derived mixed cSCCs.

A IGF1R levels in EpCAM^{high} sh-control and sh-IGF1R (2) cancer cells prior to engraftment into immunocompetent syngeneic mice. Actin was used as a loading control.

B Growth kinetics (mean ± SD) of EpCAM^{high} sh-control and sh-IGF1R (2)-derived tumors (n = 8/group). *P*-value (Repeated Measures ANOVA test). **C** Representative image of IGF1R levels in the indicated cancer cells after tumor growth. Actin was used as a loading control. **D** Percentage (mean ± SD) of EpCAM^{high}, EpCAM^{low}, and EpCAM^{neg} cancer cells generated after the engraftment of EpCAM^{high} sh-control (n = 8) and sh-IGF1R (2) (n = 8) cancer cells into immunocompetent syngeneic mice. *P*-value (unpaired two-tailed Student's *t*-test). **E-F** (**E**) Percentage (mean ± SD) of ITGAV^{+} cancer cells and (**F**) median ITGAV intensity (mean ± SD) of cancer cells in EpCAM^{high} sh-control and sh-IGF1R (2)-derived tumors. *P*-value (unpaired two-tailed Student's *t*-test). **G** Representative IF images of GFP (green)/ITGAV (red)-expressing cells in EpCAM^{high} sh-control and sh-IGF1R (1)-derived cSCCs. Nuclei stained with DAPI (blue). Scale bar: 100 µm. **H** Quantification (mean ± SD) of GFP^{+}ITGAV^{+} cancer cells per tumor area (mm^{2}) in EpCAM^{high} sh-control and sh-IGF1R (1)-derived cSCCs (n = 3/group). *P*-value (unpaired two-tailed Student's *t*-test). **I** Representative image of the induction of pSMAD3 levels after TGFβ1 treatment in the indicated cancer cells. Actin was used as a loading control. **J** Percentage (mean ± SD) of EpCAM^{high}, EpCAM^{low}, and EpCAM^{neg} cancer cells generated in TGFβ1-treated (+) and untreated (-) EpCAM^{+} plastic sh-control (n = 2) and sh-IGF1R (2) (n = 4) cancer cells. *P*-value (unpaired two-tailed Student's *t*-test). **K** Median ITGAV intensity (mean ± SD) of EpCAM^{+} plastic sh-control (n = 2) and sh-IGF1R (2) (n = 3) cancer cells treated with TGFβ1 relative to their respective untreated controls. *P*-value (unpaired two-tailed Student's *t*-test). **L** mRNA expression levels (mean ± SD) of *Igf1* and *Igf2* genes in the indicated cancer cells relative to full epithelial cancer cells (n ≥ 3/group). *P*-value (one-way ANOVA with Dunnett's test). **M** mRNA expression levels (mean ± SD) of the indicated genes in EpCAM^{high} sh-IGF1R (1 and 2) cancer cells relative to sh-control cancer cells (n = 3/group). *P*-value (unpaired two-tailed Student's *t*-test).

Supplementary Figure S6

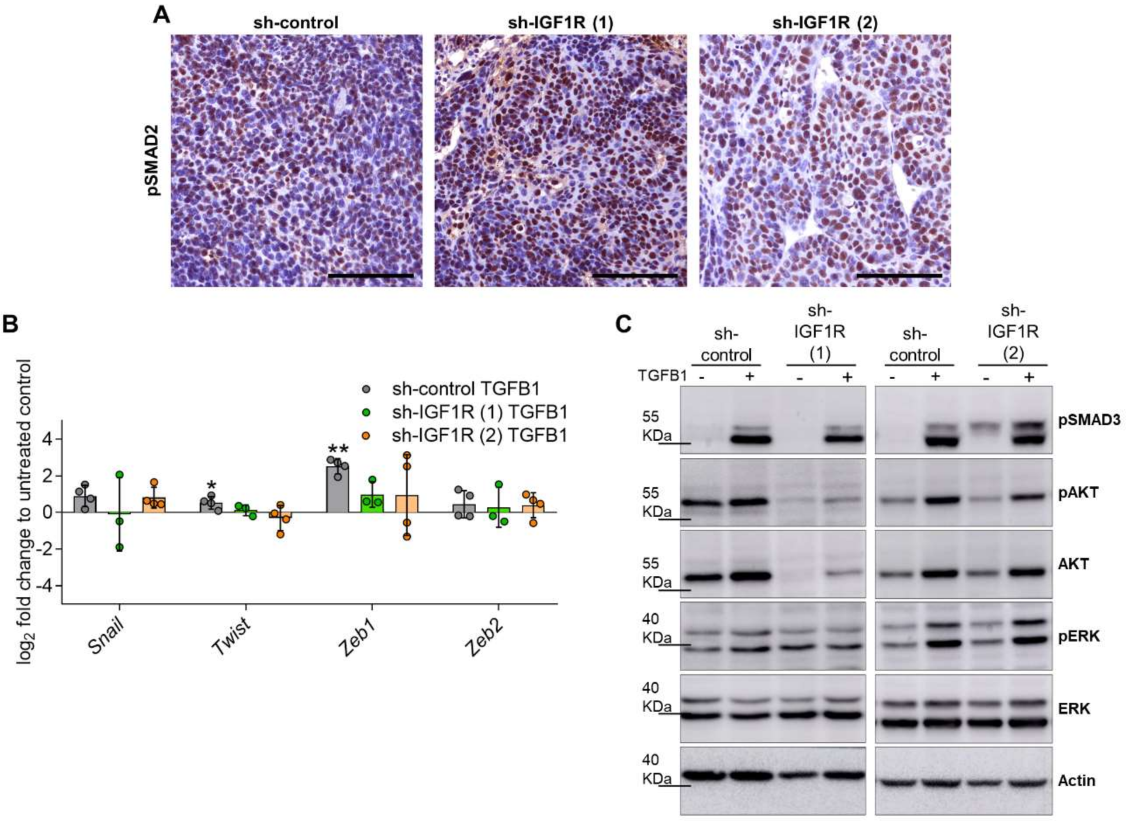

Supplementary Figure S6. IGF1R abrogation in EpCAM^{high} cancer cells blocks EMT-TFs induction without altering TGFβ signaling.

A Representative images of pSMAD2 staining in EpCAM^{high} sh-control and sh-IGF1R (1) and (2)-derived cSCCs. **B** mRNA expression levels (mean ± SD) of the EMT-TFs in EpCAM^{high} sh-control (n = 4), sh-IGF1R (1) (n = 3) and sh-IGF1R (2) (n = 4) cancer cells after 14 days of TGFβ1 treatment relative to their respective untreated controls. *P*-value (unpaired two-tailed Student's *t*-test). **C** Western blot images of the pSMAD3 (canonical TGFβ signaling), pAKT, AKT, pERK and ERK (non-canonical TGFβ signaling) levels in the indicated cells treated (+) or not (-) with TGFβ1. Actin was used as a loading control.

Supplementary Figure S7

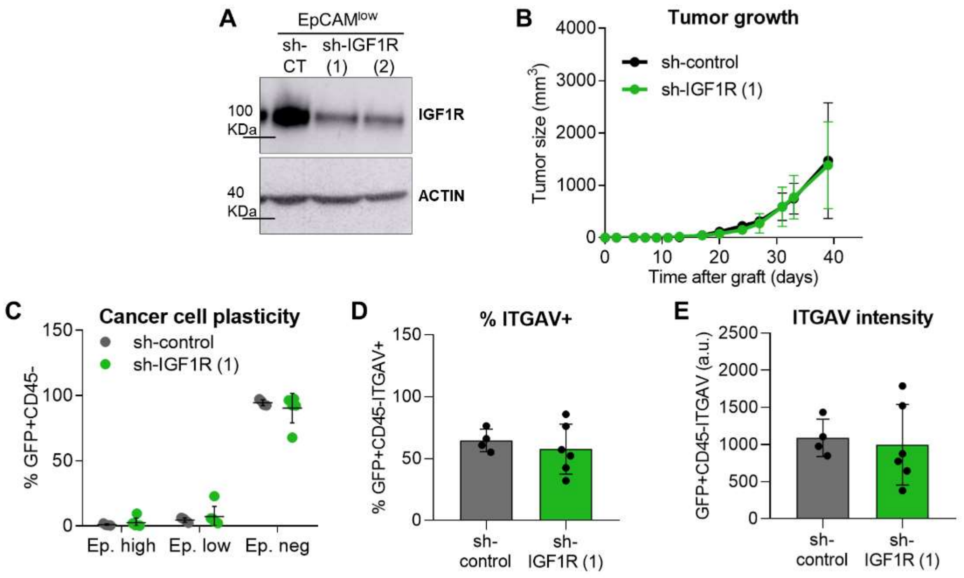

Supplementary Figure S7. IGF1R knock-down in EpCAM^{low} cancer cells does not prevent cSCC progression to the mesenchymal state.

A IGF1R levels in EpCAM^{low} sh-control and sh-IGF1R (1 and 2) cancer cells prior to engraftment into immunocompetent syngeneic mice. Actin was used as a loading control. **B** Growth kinetics (mean ± SD) of EpCAM^{low} sh-control and sh-IGF1R (1)-derived tumors (n ≥ 4/group). *P*-value (Repeated Measures ANOVA test). **C** Percentage (mean ± SD) of EpCAM^{high}, EpCAM^{low}, and EpCAM^{neg} cancer cells generated after the engraftment of EpCAM^{low} sh-control (n = 4) and sh-IGF1R (1) (n = 6) cancer cells into immunocompetent syngeneic mice. *P*-value (unpaired two-tailed Student's *t*-test). **D-E** (**D**) Percentage (mean ± SD) of ITGAV^{+} cancer cells and (**E**) median ITGAV intensity (mean ± SD) of cancer cells in EpCAM^{low} sh-control and sh-IGF1R (1)-derived tumors. *P*-value (unpaired two-tailed Student's *t*-test).

Supplementary Figure S8

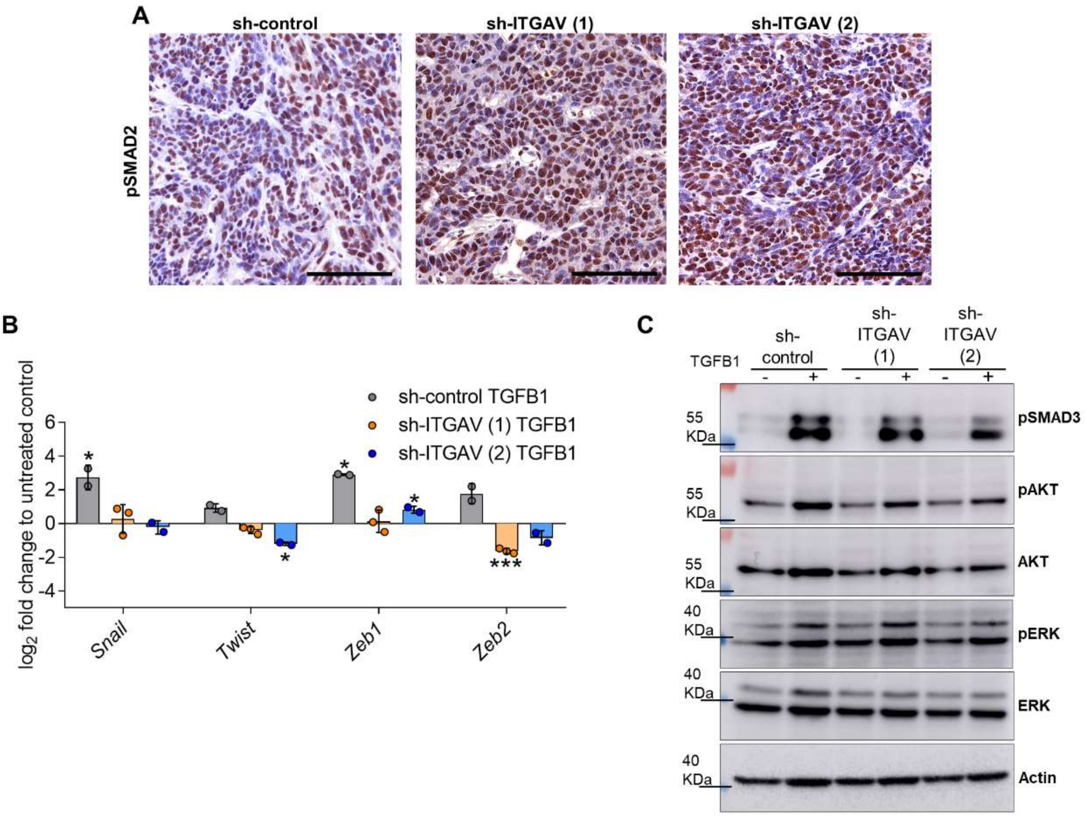

Supplementary Figure S8. ITGAV inhibition in EpCAM^{high} cancer cells reduces EMT-TFs induction without altering TGFβ signaling.

A Representative images of pSMAD2 staining in EpCAM^{high} sh-control and sh-ITGAV (1) and (2)-derived cSCCs. **B** mRNA expression levels (mean ± SD) of the EMT-TFs in EpCAM^{high} sh-control (n = 2), sh-ITGAV (1) (n = 3) and sh-ITGAV (2) (n = 2) cancer cells after 14 days of TGFβ1 treatment relative to their respective untreated controls. *P*-value (unpaired two-tailed Student's *t*-test). **C** Western blot images of the pSMAD3 (canonical TGFβ signaling), pAKT, AKT, pERK and ERK (non-canonical TGFβ signaling) levels in the indicated cells treated (+) or not (-) with TGFβ1. Actin was used as a loading control.

SUPPLEMENTARY METHODS

TGFβ *in vitro* treatment

Cancer cells were treated with 2.5 ng/μl TGFβ1 (dissolved in PBS-0.1% BSA, Peprotech, 100-21) for 14 days. Drug dissolvent was added to untreated control cancer cells. Cancer cells were isolated (≈200.000 cells/condition) by FACS-sorting from corresponding cSCCs. Each isolated primary culture was then divided into two parts (control and treated) and seeded in a plate. TGFβ1 medium was refreshed 3 times per week.

OSI-906 *in vivo* treatment

To determine the effect of the pharmacological inhibition of IGF1R pathway on the acquisition of cancer cell plasticity, isolated $GFP^{+}EpCAM^{+}$ plastic cancer cells ($1x10^4$ cells/engraftment) were mixed 1:1 with Matrigel and engrafted into immunocompetent syngeneic mice to generate mixed cSCCs. When tumors were palpable (≈ 20 mm^3 tumor volume), mice were randomly assigned to the OSI-906 (dissolved in 80% PBS + 13% PEG300 + 5% Tween-80 + 2% DMSO; HY-10191, MedChemExpress) or control (vehicle) group. Mice were administered orally 5 times per week with 30 mg/kg/dose. Tumors were excised after 15 doses (3 weeks). Mice weight was monitored weekly for possible toxic effects of the treatment.

Quantitative real-time PCR (qRT-PCR) analysis

Primers of interest: *Axl*, forward 5'- GCACAGTCTGCAAACTCCAG-3', reverse 5'-GTTTCGGCTTGAGGTTCTACC-3'; *Cdh1*, forward 5'-ATCCTCGCCCTGCTGATT-3', reverse 5'-ACCACCGTTCTCCTCCGTA-3'; *Cldn7*, forward 5'-GCCAGGTCAAAAATCAAGTTTACCA-3', reverse 5'-

TTGGACTTGGGGTAAGAGCG-3’; *dNp63*, forward 5’-GTACCTGGAAAACAATGCCCAG-3’, reverse 5’-CGCTATTCTGTGCGTGGTCTG-3’; *Epcam,* forward 5’-CCGCGGCTCAGAGAGACT-3’, reverse 5’-AGGAAGTACACTGGCATTCACC-3’; *Gapdh*, forward 5’-AGGTCGGTGTGAACGGATTTG-3’, reverse 5’-TGTAGACCATGTAGTTGAGGTCA-3’; *Grhl1,* forward 5’-CCTTCACGTGGGACATCAAT-3’, reverse 5’-AGCCCTTCACACCCTTCTG-3’; *Grhl2*, forward 5’-GACAACAAATGCTTCCGACA-3’, reverse 5’-GCTGCTCATCTCGGTTTTTG-3’; *Igf1*, forward 5’-GGACCAGAGACCCTTTGCGGG-3’, reverse 5’-GGCTGCTTTTGTAGGCTTCAGTGG-3’; I*gf2*, forward 5’-GGGAAGTCGATGTTGGTGCT-3’, reverse 5’-AGGCCTGCTGAAGTAGAAGC-3’; *Igf1r*, forward 5’-GCTTCTGTGAACCCCGAGTATTT-3’, reverse 5’-TGGTGATCTTCTCTCGAGCTACCT-3’; *Itgav,* forward 5’-TTCACACTTTGGGCTGTGGA-3’, reverse 5’-TTCACGTACAGGATTGCGCT-3’; *Itgb3,* forward 5’-AGTGGCCGGGACAACTCT-3’, reverse 5’-TTGGACTCTCCAACAACAACGC-3’; *Krt7,* forward 5’-GCGGAGATGAACCGCTCTAT-3’, reverse 5’-TCTAACTTGGCACGCTGGTT-3’; *Krt14,* forward 5’-GGCCCAGATCCAGGAGATGAT-3’, reverse 5’-CAGGGGCTCTTCCAGCAGTATC-3’; *Ovol1,* forward 5’-CTCCACGTGCAAGAGGAACT-3’, reverse 5’-CTCTGGTTCCCGGTAGGG-3’; *Ovol2,* forward 5’-GCCAGGTCAAAAATCAAGTTTACCA-3’, reverse 5’-AGCTCTTGCCACAAAGGTCA-3’; *Ppia,* forward 5’-GTTCATGCCTTCTTTCACCTTCCC-3’, reverse 5’-CAAATGCTGGACCAAACACAAACG-3’; *Snail,* forward 5’-CTTGTGTCTGCACGACCTGT-3’, reverse 5’-AGTGGGAGCAGGAGAATGG-3’;

Sox4, forward 5'-CCAGCAAGAAAAGAAGCCAA-3', reverse 5'-TGACCATGAGGCAAAATCAA-3'; *Spp1,* forward 5'-TCCCTCCCGGTGAAAGTGA-3', reverse 5'-TCTGTGGCGCAAGGAGATTC-3'; *Tgfb1*, forward 5'-GTCAGCAGCCGGTTACCA-3', reverse 5'-TGGAGCAACATGTGGAACTC-3'; *Tgfb2*, forward 5'-AACTCCATAGATATGGGGATGC-3', reverse 5'-AATGTGCAGGATAATTGCTGC-3'; *Tnc,* forward 5'-TGAACGGACTGCCCACATCT-3', reverse 5'-CTTCCGGTTCAGCTTCTGTGGTA-3'; *Twist,* forward 5'-AGCTACGCCTTCTCCGTCT-3', reverse 5'-TCCTTCTCTGGAAACAATGACA-3'; *Vcam1,* forward 5'-TTTTCACGTGGGGCACAAAG-3', reverse 5'-AGCTTGAGAGACTGCAAACA-3'; *Vim,* forward 5'-AGAGAGAGGAAGCCGAAAGC-3', reverse 5'-TCCACTTTCCGTTCAAGGTC-3'; *Zeb1,* forward 5'-GCCAGCAGTCATGATGAAAA-3', reverse 5'-TATCACAATACGGGCAGGTG-3'; and *Zeb2,* forward 5'-TCTTATCAATGAAGCAGCCG-3', reverse 5'-TGCGTCCACTACGTTGTCAT-3'.

Microarray analysis

Summary expression values for all probe sets were pre-processed and normalized using the RMA function as implemented in the affy package from Bioconductor [1]. Differential expression analysis was performed using the linear modeling method implemented in the Limma package. Multiple testing was corrected by the Benjamini-Hochberg false discovery rate (FDR) method. Differentially expressed genes were defined as those with a ≥ 1 $\log_2$-FC and an FDR *P*-value of $< 5\%$. Genes were ranked according to $\log_2$-FC. R packages ClusterProfiler v4.10.0 [2] and MSigDBr v7.5.1 were used to perform gene set enrichment analysis for Gene Ontology, Hallmark [3], KEGG and ImmuneSigDB signatures, using an FDR *P*-value of < 0.05 and *q*-value of < 0.10.

Phosphoproteomic analysis

Three biological replicates of full epithelial, EpCAM^{+} plastic, and EpCAM^{neg} cancer cells were grown *in vitro* to 70-80% confluency, washed with PBS, scrapped into the lysis buffer (9 M urea, 20 mM HEPES pH 8.0, 1 mM sodium orthovanadate, 2.5 mM sodium pyrophosphate, 1 mM β-glycerophosphate), and sonicated using a Branson high-intensity cuphorn sonicator (3 cycles of 30 s). Cell lysates were reduced by incubation in 45 mM DTT for 30 min at 55ºC and alkylated in 110 mM iodoacetamide for 15 min at room temperature in the dark. Subsequently, cell lysates were diluted to 2 M urea using 20 mM HEPES pH 8.0 and digested overnight at room temperature with 5 µg/ml trypsin (Promega, V542). After acidifying the digests by adding trifluoroacetic acid (TFA) to a final concentration of 1%, tryptic digests were desalted and purified on Sep-Pak C18 columns using a vacuum system, eluted in 50% acetonitrile (ACN) and 0.1% TFA, and lyophilized for at least 48h. For global phosphopeptide analysis, desalted peptides were enriched with titanium dioxide (TiO_2) beads, using aliphatic hydroxy-acid modified metal oxide chromatography (MOAC). For specific enrichment of phosphotyrosine (pTyr)-containing peptides, lyophilized peptides were dissolved in 700 µl immunoprecipitation buffer (20 mM Tris-HCl pH 7.2, 10 mM sodium phosphate, 50 mM NaCl) and transferred at 4ºC to a tube containing 20 µl of a 50% (v/v) slurry of agarose beads harboring P-Tyr-1000 anti-phosphotyrosine monoclonal antibodies (Cell Signaling Technologies, 8803). Following a 2h incubation at 4ºC on a rotator, beads were washed twice with cold PBS, and 3 times with cold Milli-Q water. Bound peptides were eluted with a total of 50 µl 0.15% TFA in two steps. Peptides were desalted with custom-made C18 stage tips and eluted in 0.1% TFA. Then, peptides were speed-vac dried, solubilized in 20 µl loading solvent (4% ACN in 0.5% TFA), and samples were subjected to LC-MS/MS analysis. Peptides were separated by an Ultimate 3000 nanoLC-MS/MS system (Dionex LC-Packings, The Netherlands), and eluting peptides were ionized at a potential of +2 kV and

introduced into a Q Exactive mass spectrometer (Thermo Fisher, Bremen, Germany). MS/MS spectra were searched separately against the Swissprot mouse FASTA file (canonical and isoforms 2017, 25052 entries) using MaxQuant 1.5.4.1. Phosphopeptide identifications were propagated across samples using the 'match between runs' option checked. Phosphopeptides were relatively quantified by their extracted ion intensities ('Intensity' in MaxQuant). For each sample, the phosphopeptide intensities were normalized on the median intensity of all identified peptides in the sample. Overall kinase phosphorylation was calculated by summing all identified phosphopeptides MS/MS spectra for a kinase (spectral-counting). Normalization, statistical testing, and cluster analysis of differential phosphopeptides were performed in R Studio. Their in-house developed kinase-focused pipeline based on kinase-centered phosphorylation analysis (INKA), the substrate-based motif analysis (NetworKIN) and the PhosphositePlus database were used for integrative inferred kinase activity scoring.

SUPPLEMENTARY REFERENCES

1. Gautier L, Cope L, Bolstad BM, Irizarry RA. affy—analysis of Affymetrix GeneChip data at the probe level. Bioinformatics. 2004;20:307–15.

2. Wu T, Hu E, Xu S, Chen M, Guo P, Dai Z, et al. clusterProfiler 4.0: A universal enrichment tool for interpreting omics data. Innovation [Internet]. 2021 [cited 2023 Nov 3];2. Available from: https://www.cell.com/the-innovation/abstract/S2666-6758(21)00066-7

3. Liberzon A, Birger C, Thorvaldsdóttir H, Ghandi M, Mesirov JP, Tamayo P. The Molecular Signatures Database Hallmark Gene Set Collection. cels. 2015;1:417–25.
